# Intracellular HMGB1 as a novel tumor suppressor of pancreatic cancer

**DOI:** 10.1038/cr.2017.51

**Published:** 2017-04-04

**Authors:** Rui Kang, Yangchun Xie, Qiuhong Zhang, Wen Hou, Qingping Jiang, Shan Zhu, Jinbao Liu, Dexing Zeng, Haichao Wang, David L Bartlett, Timothy R Billiar, Herbert J Zeh, Michael T Lotze, Daolin Tang

**Affiliations:** 1 The Third Affiliated Hospital, Center for DAMP Biology, Key Laboratory for Major Obstetric Diseases of Guangdong Province, Key Laboratory of Reproduction and Genetics of Guangdong Higher Education Institutes, Protein Modification and Degradation Laboratory, Guangzhou Medical University, Guangzhou, Guangdong 510510, China; 2 Department of Medicine, University of Pittsburgh, Pittsburgh, PA 15219, USA; 3 Laboratory of Emergency Medicine, The Feinstein Institute for Medical Research, Manhasset, NY 11030, USA; 4 Department of Surgery, Hillman Cancer Center, University of Pittsburgh, Pittsburgh, PA 15219, USA

**Keywords:** HMGB1, RAGE, histone, K-Ras, pancreatic cancer

## Abstract

Pancreatic ductal adenocarcinoma (PDAC) driven by oncogenic K-Ras remains among the most lethal human cancers despite recent advances in modern medicine. The pathogenesis of PDAC is partly attributable to intrinsic chromosome instability and extrinsic inflammation activation. However, the molecular link between these two events in pancreatic tumorigenesis has not yet been fully established. Here, we show that intracellular high mobility group box 1 (HMGB1) remarkably suppresses oncogenic K-Ras-driven pancreatic tumorigenesis by inhibiting chromosome instability-mediated pro-inflammatory nucleosome release. Conditional genetic ablation of either single or both alleles of HMGB1 in the pancreas renders mice extremely sensitive to oncogenic K-Ras-driven initiation of precursor lesions at birth, including pancreatic intraepithelial neoplasms, intraductal papillary mucinous neoplasms, and mucinous cystic neoplasms. Loss of HMGB1 in the pancreas is associated with oxidative DNA damage and chromosomal instability characterized by chromosome rearrangements and telomere abnormalities. These lead to inflammatory nucleosome release and propagate K-Ras-driven pancreatic tumorigenesis. Extracellular nucleosomes promote interleukin 6 (IL-6) secretion by infiltrating macrophages/neutrophils and enhance oncogenic K-Ras signaling activation in pancreatic lesions. Neutralizing antibodies to IL-6 or histone H3 or knockout of the receptor for advanced glycation end products all limit K-Ras signaling activation, prevent cancer development and metastasis/invasion, and prolong animal survival in Pdx1-Cre;K-Ras^G12D/+^;Hmgb1^−/−^ mice. Pharmacological inhibition of HMGB1 loss by glycyrrhizin limits oncogenic K-Ras-driven tumorigenesis in mice under inflammatory conditions. Diminished nuclear and total cellular expression of HMGB1 in PDAC patients correlates with poor overall survival, supporting intracellular HMGB1 as a novel tumor suppressor with prognostic and therapeutic relevance in PDAC.

## Introduction

Pancreatic ductal adenocarcinoma (PDAC) represents over 90% of all pancreatic malignancies. PDAC has the highest mortality rate of solid organ cancers, with only a 7% five-year survival rate^1^. PDAC shares some characteristics with other solid malignancies such as mutations affecting common signaling pathways, tumor heterogeneity, development of invasive malignancy from precursor lesions, inherited forms of the disease, and common environmental risk factors. However, unique obstacles have hindered the fight against PDAC. These include: 1) difficulties in diagnosis at an early stage in the disease because of the lack of specific symptoms or biomarkers and the anatomical location of the pancreas; 2) metastatic spread when the primary tumor is too small to detect using current methods; 3) dynamic interaction of the tumor with stromal cells, creating dense fibrous tissue around the tumor that contributes to therapeutic resistance; 4) avoidance of recognition and attack by the immune system, and 5) the small percentage of patients for whom curative surgery is a feasible option^2,3^. The need to further improve our understanding of pancreatic cancer biology, identify biomarkers, develop targeted therapies, and select eligible patient subgroups is unquestionably urgent.

Identification of clinically meaningful approaches relies heavily on the availability of preclinical models that recapitulate the key morphological and molecular features of the disease and offer high predictive value for clinically useful diagnostic and therapeutic interventions^4,5^. In particular, genetically engineered mouse models of PDAC have progressively improved in technical sophistication and, recapitulating features of human PDAC, and have had a measureable impact on our knowledge of tumorigenesis^6,7,8^. PDAC is characterized by a high frequency (> 95%) of activation of K-Ras mutations (especially G12D)^9,10^ and progresses from non-invasive pancreatic lesions that include pancreatic intraepithelial neoplasias (PanINs), intraductal papillary mucinous neoplasms (IPMNs), and mucinous cystic neoplasms (MCNs)^11^. Although oncogenic K-Ras plays a central role in controlling PDAC initiation and progression, the ability of mutant K-Ras to drive PDAC was not successfully investigated until the generation of mice with a *Cre*-inducible conditional allele (*Pdx1-Cre;K-Ras^G12D/+^*, termed KC mice) targeting the endogenous *K-Ras* locus^12^. These KC mice develop lesions that slowly progress into advanced PDAC and have a median survival of 15 months^12^, suggesting that K-Ras activation is a tumor-initiating event that requires other elements that accelerate PDAC progression. Further understanding of K-Ras signaling and regulation may translate into improved treatments for pancreatic cancer.

High-mobility group box 1 (HMGB1) was first discovered as one of a group of chromatin-associated proteins with high acidic and basic amino acid content^13^. It is a highly-conserved protein with > 99% amino acid identity between murine and human molecules. Structurally, HMGB1 protein contains two homologous DNA-binding domains (termed A and B boxes, each 75 amino acids in length) with a negatively charged C-terminal region. Under normal conditions, most HMGB1 is localized in the nucleus to relax structural constraints within the nucleosome. Nuclear HMGB1 can bind to and bend DNA to control gene transcription, DNA repair, chromatin remodeling, and V(D)J recombination^14,15^. For example, HMGB1 is a transcriptional cofactor of p53, p73, the retinoblastoma protein, NF-κB, and nuclear hormone receptors including the estrogen receptor^14,15^. HMGB1 is also recognized as a damage-associated molecular pattern (DAMP) during cell death, inflammation, and encounter with various environmental stressors^14,15,16,17^. To act as a DAMP and danger signal, HMGB1 is required to be released by two different ways: active secretion from living immune cells or passive release from dead, dying, and injured cells. Dysfunction of intracellular and extracellular HMGB1 has been implicated in multiple human diseases or diverse pathologies including infections, cancer, neurodegeneration, aging, and heart disease^18^. In particular, HMGB1 was found to be a therapeutic target for tissue injury-mediated sterile inflammation and pathogen-mediated infection^19^. Interestingly, HMGB1 has dual roles in cancer development, progression, and therapy^20,21^. In many cases, extracellular HMGB1 acts as a pro-tumor protein due to its cytokine, chemokine, and growth factor activity, whereas intracellular HMGB1 promotes drug resistance due to its proautophagic activity^22,23^. However, the functional contribution of intracellular HMGB1 to tumorigenesis was previously unknown. In this study, we examined the impact of HMGB1 deficiency in pancreata on mutant K-Ras-driven initiation and progression of PDAC in mice, and investigated the underlying molecular mechanism as well as potential clinical significance. Our study indicates that intracellular HMGB1 is a novel tumor suppressor of PDAC by sustaining chromosome stability and limiting pro-inflammatory nucleosome release and activity.

## Results

### HMGB1 depletion accelerates initiation and progression of K-Ras-driven PDAC

**Figure 1:**
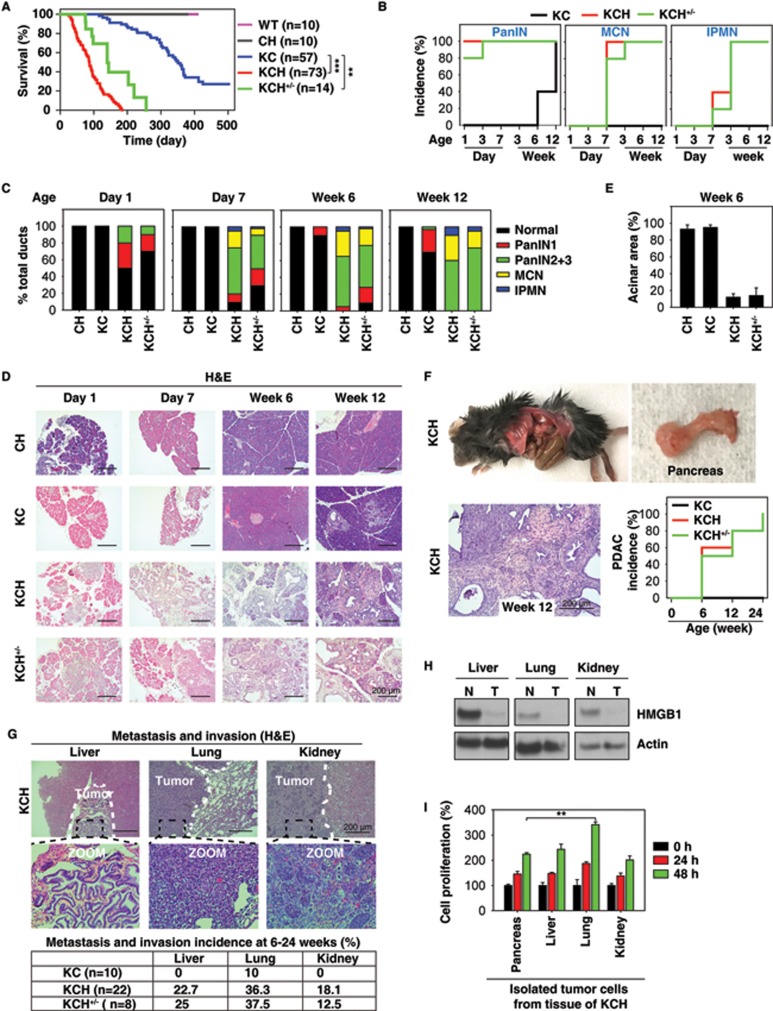
HMGB1 depletion accelerates initiation and progression of K-Ras-driven PDAC. **(A)** Kaplan-Meier survival analysis was performed in wild type (WT), CH (*Pdx1-Cre;Hmgb1*^−/−^), KC (*Pdx1-Cre;K-Ras^G12D/+^*;*Hmgb1*^+/+^), KCH (*Pdx1-Cre;K-Ras^G12D/+^*;*Hmgb1*^−/−^), and KCH^+/^^−^ (*Pdx1-Cre;K-Ras^G12D/+^*;*Hmgb1^+/−^*) mice (^**^P < 0.01, ^***^P < 0.001, log-rank test). **(B)** Incidence of pancreatic lesions, including pancreatic intraepithelial neoplasms (PanINs), intraductal papillary mucinous neoplasms (IPMNs), and mucinous cystic neoplasms (MCNs) in KC, KCH, and KCH^+/−^ mice at indicated ages (*n* = 5 mice/genotype /age). **(C)** Percentages of ductal structures exhibiting normal morphology and indicated neoplastic ducts in KC, CH, KCH, and KCH^+/−^ mice (*n* = 5 mice/genotype /age). **(D)** Representative histologic progression of pancreata in KC, CH, KCH, and KCH^+/−^ mice at indicated ages shown by hematoxylin and eosin (H&E) staining (high resolution images shown in Supplementary information, Figure S2). **(E)** Percentages of intact acinar structures in pancreata from KC, CH, KCH, and KCH^+/−^ mice at six weeks of age (*n* = 5 mice/genotype). **(F)** Incidence of PDAC in pancreata from KC, KCH, and KCH^+/−^ mice (*n* = 5 mice/genotype/age). Representative samples H&E-stained for PDAC with stromal structure shown in right panel. **(G)** Incidence of tumor metastasis/invasion in KC, KCH, and KCH^+/−^ mice at six to 24 weeks of age. Representative samples H&E-stained for tumor metastasis/invasion to the liver, lung, and kidney shown in upper panels. **(H)** Western blot analysis of HMGB1 expression in isolated tumor (T) or normal (N) tissue from KCH mice. **(I)** Cell proliferation analysis of indicated isolated tumor cells from KCH mice (*n* = 3, ^**^P < 0.01, unpaired *t*-test).

We created mice with conditional knockout of HMGB1 in the pancreas (*Pdx1-Cre;Hmgb1*^−/−^, termed CH mice) using *Cre/loxP* strategies and reported that CH mice exhibit normal pancreatic development and function under physiological conditions, but are more sensitive to experimental pancreatitis under pathological conditions^24^. Given that pancreatitis is considered one of the major risk factors for PDAC^25^, we postulated that HMGB1 would regulate K-Ras-driven pancreatic tumorigenesis and generated mice conditionally defective in both *K-Ras* and *Hmgb1* in the pancreatic tissue (*Pdx1-Cre;K-Ras^G12D/+^*;*Hmgb1*^−/−^, termed KCH mice; Supplementary information, Figure S1). These KCH mice were born at normal Mendelian frequency, exhibiting substantial weight loss and even sudden death at ∼3-6 weeks of age, resulting in a median survival of approximately three months (Figure 1A). These findings recapitulate those frequently found in humans with PDAC at presentation or progression. The average survival time of heterozygous HMGB1 knockout mice (*Pdx1-Cre;K-Ras^G12D/+^*;*Hmgb1*^+/−^; termed KCH^+/−^ mice) is slightly longer (4.9 months, Figure 1A), but with similar PDAC phenotypes observed in KCH mice (*vide infra*). These findings indicate that HMGB1 is involved in K-Ras-driven pancreatic cancer development.

We next assessed the impact of HMGB1 deletion in mice expressing *K-Ras^G12D/+^* on development of precursor lesions and PDAC. In line with a previous report^12^, control KC mice displayed no IPMNs or MCNs, only low grade PanINs (PanIN-1) at three months of age (Figure 1B and 1C) but high grade PanINs (PanIN-2 and -3) at nine months within an otherwise normal pancreas (data not shown). In contrast, pancreata from KCH mice exhibited 100% low and high grade PanINs at birth (Figure 1B and 1C), occupying the entire pancreas and resulting in almost complete loss of normal pancreatic tissue as early as day 7 (Figure 1D and Supplementary information, Figure S2). KCH^+/−^ mice also exhibited low and high grade PanINs on day 3 (Figure 1B-D). In addition to PanINs, cystic lesions, including IPMNs and MCNs, also appeared in pancreata from KCH and KCH^+/−^ mice (Figure 1B-1D and Supplementary information, Figure S3), as frequently observed in human patients^26^. The percentage of normal pancreatic acinar cells was remarkably reduced in KCH and KCH^+/−^ mice (Figure 1D and 1E and Supplementary information, Figure S2) with decreased exocrine function (Supplementary information, Figure S4A and S4B). Blood glucose levels are tightly controlled by regulation of insulin release from pancreatic beta-cells. We observed that blood glucose levels were increased (Supplementary information, Figure S4C) whereas serum insulin levels were decreased (Supplementary information, Figure S4D) in KCH mice due to the loss of pancreatic islet beta-cells (Supplementary information, Figure S4E and S4F). Unlike KCH mice, CH mice had normal exocrine and endocrine function, as demonstrated in our previous study^24^. These findings indicate that loss of HMGB1 is dispensable for normal pancreatic development, but promotes pancreatic function insufficiency during K-Ras-driven tumor development.

It is clear that pancreatic lesions are mainly derived from acinar cells undergoing acinar to ductal metaplasia (ADM), an event usually induced by pancreatitis^27^. We therefore hypothesized that loss of HMGB1 may promote ADM in K-Ras-driven pancreatic tumorigenesis. Indeed, KCH and KCH^+/−^ mice exhibited significant ADM, characterized by colocalization of amylase (an acinar cell marker) and cytokeratin 19 (CK19, a ductal cell marker) within lesions (Supplementary information, Figure S5A). The expression of sex determining region Y-box 9 (SOX9), a core regulator of ADM and initiation of PDAC, has been identified in PanINs, IPMNs, and MCNs in mouse and human pancreatic cancer specimens^28^. Consistently, the expression of SOX9 was elevated in KCH and KCH^+/−^ mice (Supplementary information, Figure S5B). Thus, loss of HMGB1 is an accelerating event for the development of these various premalignant neoplasms (e.g., ADM and SOX9 expression) of PDAC.

We explored further the role of HMGB1 in PDAC development. In KC mice, the onset of PDAC usually occurs beyond 12 months^12^. In contrast, KCH mice between six and 24 weeks old exhibited progression to either locally invasive PDAC (Figure 1F and Supplementary information, Figure S3) or PDAC with increased metastatic or invasion capacity to the liver, lung, and kidney (Figure 1G and Supplementary information, Table S1). Tumors in the liver (but not lung or kidney) shared a similar morphology to pancreatic tumor in KCH mice (Figure 1G). Western blot analysis confirmed loss of HMGB1 protein expression in metastatic or invading tumor from liver, lung, and kidney (Figure 1H). Moreover, increased cell proliferation was observed in isolated metastatic or invading tumor cells from lung (but not liver or kidney) compared to HMGB1-deficient primary pancreatic tumor cells (Figure 1I). Further characterization of KCH and KCH^+/−^ mice with immunofluorescence microscopy of pancreata showed that the loss of HMGB1 increased ductal lesions, as determined by CK19 positivity (Supplementary information, Figure S6A) and mucin expression based on Alcian blue staining (Supplementary information, Figure S6B). Notably, pancreata from KCH and KCH^+/−^ mice also showed abundant stromal components (a hallmark of PDAC), as determined by vimentin positivity (Supplementary information, Figure S7A) and Masson's trichrome staining (Supplementary information, Figure S7B). Ki-67 (Supplementary information, Figure S8A) staining of the ductal epithelium showed that loss of HMGB1 enhanced K-Ras-induced cellular proliferation. Consistent with the increased matrix metalloproteinase-7 (MMP7) expression observed in pre-invasive and invasive human pancreatic cancer specimens^29^, we also found elevated expression of MMP7 in ductal lesions of KCH and KCH^+/−^ mice (Supplementary information, Figure S8B). Collectively, this changed histology confirmed that loss of HMGB1 accelerates K-Ras-driven pancreatic tumorigenesis and development.

### Oxidative DNA damage promotes pancreatic tumorigenesis in KCH mice

**Figure 2:**
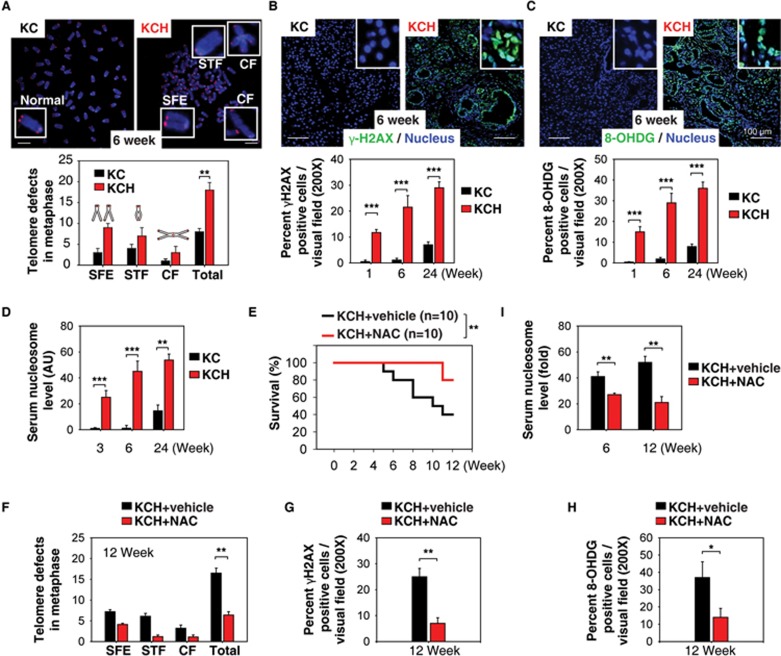
Oxidative DNA damage promotes pancreatic tumorigenesis in KCH mice. **(A)** Percentage of abnormal telomeres in ductal cells from KC and KCH mice at six weeks of age (*n* = 5 mice/genotype). CF = chromosome fusions and concatenation; SFE = telomere signal-free end; STF = sister telomere fusion. Total = CF + SFE + STF. **(B-C)** Immunohistochemical staining of DNA damage marker (γ-H2AX) and oxidative DNA damage marker (8-OHDG) in KC and KCH mice. **(D)** ELISA analysis of serum nucleosome levels in KC and KCH mice (*n* = 5 mice/genotype/age). **(E-I)** Antioxidant N-acetylcysteine (NAC, 300 mg/kg i.p., five times per week, started at 4 weeks of age for four weeks) treatment prolonged survival in KCH mice (**E**, ^**^P < 0.01, log-rank test) with decreased telomere defects **(F)**, histone H2AX phosphorylation **(G)**, oxidative DNA damage **(H)**, and serum nucleosome levels **(I)**. ^*^P < 0.05, ^**^P< 0.01 (*n* = 5 mice/genotype, unpaired *t*-test).

Chromosomal instability is an intrinsic pathway responsible for mutagenesis and carcinogenesis^30^. Despite its widespread prevalence, knowledge of the mechanisms and contributions of chromosomal instability in cancer remains largely unknown. As HMGB1 acts as a DNA chaperone, influencing multiple processes in chromatin such as gene transcription and nucleosome assembly^31,32^, we postulated that HMGB1 regulates chromosomal stability and the DNA damage response in pancreatic tumorigenesis. Telomere analysis by fluorescence *in situ* hybridization showed an increase in morphological defects (including chromosome fusion and concatenation, apparent loss of telomeres, and sister telomere fusion) in ductal cells from KCH mice compared with KC mice (Figure 2A). Levels of histone H2AX phosphorylation (γ-H2AX, a marker for DNA damage^33^; Figure 2B) and 8-hydroxyguanine (8-OHDG, a marker of oxidative DNA damage^34^; Figure 2C) were also upregulated in the ductal lesions of KCH mice. These were accompanied by abnormal expression of genes involved in regulating the DNA damage response (Supplementary information, Figure S9A) and telomere maintenance (Supplementary information, Figure S9B) in KCH mice compared with KC mice. Thus, loss of HMGB1 in the pancreas accelerates oxidative DNA damage and chromosome instability in K-Ras-driven pancreatic tumorigenesis.

Pancreatitis is an extrinsic pathway contributing to the pathogenesis of PDAC, and inflammatory mediators exert pleiotropic effects in the development of PDAC^35^. Extracellular nucleosomes including histone and DNA have been suggested to be inflammatory mediators for cell death^36^. We previously demonstrated that loss of HMGB1 in the pancreas increases pro-inflammatory nucleosome release in experimental pancreatitis by oxidative injury^24^. Accordingly, serum nucleosome levels were elevated in KCH mice compared with KC mice at 1 to 24 weeks of age (Figure 2D). Furthermore, we found that treating 4-week-old KCH mice with the antioxidant N-acetylcysteine (NAC) for 4 weeks significantly prolonged animal survival (Figure 2E) with decreased telomere defects (Figure 2F), histone H2AX phosphorylation (Figure 2G), oxidative DNA damage (Figure 2H) and serum nucleosome levels (Figure 2I). These findings suggest that oxidative DNA damage-mediated nucleosome release contributes to PDAC in KCH mice.

### Extracellular histone promotes pancreatic tumorigenesis in KCH mice

**Figure 3:**
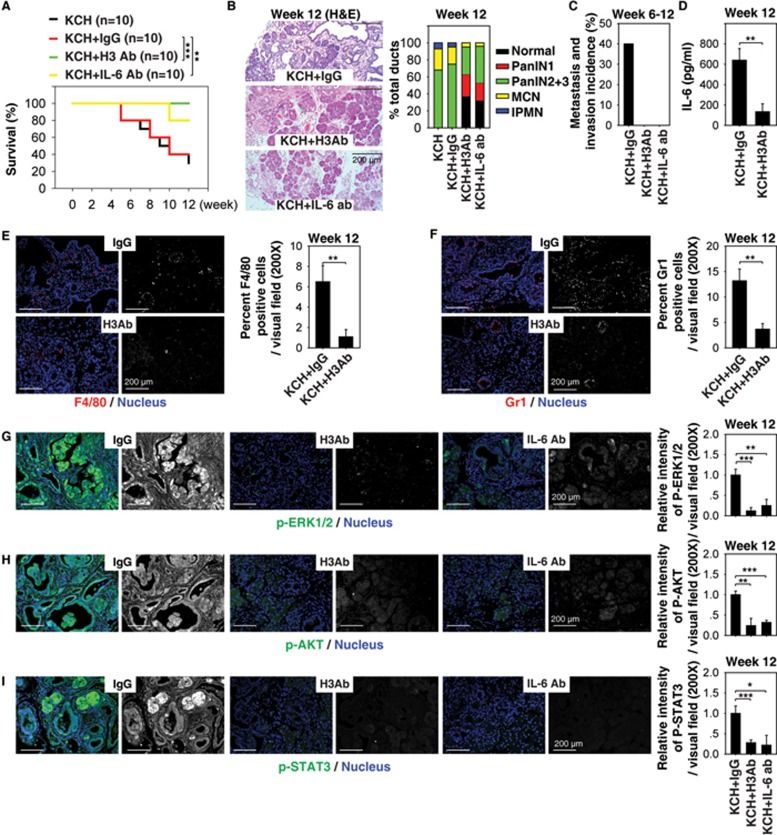
Extracellular histone promotes pancreatic tumorigenesis in KCH mice. **(A)** H3 Ab or IL-6 Ab treatment (10 mg/kg i.p., twice per week, started at four weeks of age for four weeks) prolonged survival in KCH mice at 12 weeks of age (*n* = 10 mice/treatment, ^**^P < 0.01, ^***^P < 0.001, log-rank test). The median survival of untreated, IgG-treated, H3 Ab-treated, and IL-6 treated KCH mice were nine, nine, 12, and 12 weeks, respectively, in this setting. **(B-I)** In parallel, pancreatic lesion formation **(B)**, incidence of tumor metastasis/invasion **(C)**, serum IL-6 level **(D)**, percentage of tumor infiltration of macrophages **(E)** and neutrophils **(F)**, and relative expression of p-ERK1/2 **(G)**, p-AKT **(H)**, and p-STAT3 **(I)** in the pancreas were assayed. Graphs show means ± sem, ^*^P < 0.05, ^**^P < 0.01, ^***^P < 0.001 (*n* = 5 mice/treatment, unpaired *t*-test).

Next, we investigated whether suppression of extracellular nucleosome activity could impact PDAC development in KCH mice. As histone H3 is one of the five main histone proteins in the nucleosome, we tested the effects of neutralizing blood nucleosome activity with an anti-H3 antibody upon tumor development in KCH mice. Notably, this treatment resulted in prolonged animal survival (Figure 3A), decreased pancreatic lesion formation (Figure 3B), increased normal acinar structures (Figure 3B), and reduced metastasis/invasion to the liver, lung, and kidney (Figure 3C). These findings indicate that circulating nucleosomes promote pancreatic tumorigenesis in KCH mice.

We and others have previously reported that interleukin (IL-6) is a key PDAC-associated proinflammatory cytokine promoting K-Ras and activating the STAT3 (signal transducer and activator of transcription 3) signaling in PDAC^37,38,39,40,41^. Constitutive activation of STAT3 is implicated in stem cell self-renewal, cancer cell survival, and inflammation in PDAC. We next sought to determine whether the IL-6-STAT3 pathway is affected by circulating nucleosome in KCH mice. We found that the serum IL-6 level (Figure 3D) was also reduced in KCH mice following treatment with anti-H3 antibody. Analysis of tumor infiltrations of myeloid cells such as macrophages (Figure 3E) and neutrophils (Figure 3F) by staining for F4/80 or Gr-1 antibody, respectively, revealed that these major cellular sources of IL-6 were decreased in KCH mice following treatment with anti-H3 antibody.

Further characterization of the activation of phospho-extracellular signal-regulated kinase-1/2 (p-ERK1/2) (Figure 3G), phospho-V-Akt murine thymoma viral oncogene homolog (p-AKT) (Figure 3H), and p-STAT3 (Figure 3I) in the pancreas revealed striking differences following anti-H3 antibody treatment. The increased levels of p-ERK1/2 (Figure 3G), p-AKT (Figure 3H), and p-STAT3 (Figure 3I) in the pancreata (especially tumor lesions) of KCH mice were suppressed by treatment with anti-H3 antibody. Accordingly, treatment with anti-IL-6-neutralizing antibody also increased animal survival (Figure 3A) and decreased levels of p-ERK1/2 (Figure 3G), p-AKT (Figure 3H), and p-STAT3 (Figure 3I) in the pancreata of KCH mice. These findings suggest that extracellular nucleosome activity contributes to the inflammatory response, sustaining IL-6/STAT3 signaling within the pancreatic tumor microenvironment.

### Deletion of RAGE protects against pancreatic tumorigenesis in KCH mice

Several receptors, including the receptor for advanced glycation end products (RAGE)^42^, Toll like receptor (TLR)-2^43^, TLR4^43^, and TLR9^44^, mediate the biological activity of extracellular nuclear DAMP in individual cell types. Knockout of RAGE or TLR9 inhibits development of pancreatic lesions and progression to PDAC in KC mice^39,45^. Expression of protein (Supplementary information, Figure S10A and S10B) and mRNA (Supplementary information, Figure S10C) of RAGE and TLR9 (but not TLR2 and TLR4) was increased in the pancreas of KCH mice compared with KC mice, indicating a potential role of RAGE and TLR9 in pancreatic tumorigenesis.

**Figure 4:**
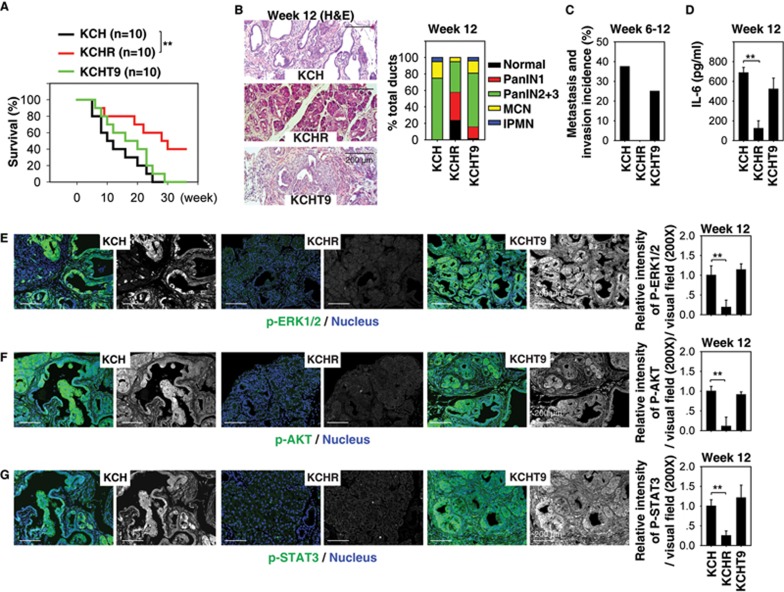
Deletion of RAGE protects against pancreatic tumorigenesis in KCH mice. **(A)** Kaplan-Meier survival analysis was performed in KCH (*Pdx1-Cre;K-Ras^G12D/+^*;*Hmgb1*^−/−^), KCHR (*Pdx1-Cre;K-Ras^G12D/+^*;*Hmgb1*^−/−^;*Rage*^−/−^), and KCHT9 (*Pdx1-Cre;K-Ras^G12D/+^*;*Hmgb1*^−/−^;*Tlr9*^−/−^) mice (*n* = 5 mice/treatment, ^**^P < 0.01, log-rank test). **(B-G)** In parallel, pancreatic lesion formation **(B)**, tumor metastasis/invasion incidence **(C)**, serum IL-6 level **(D)**, and relative expressions of p-ERK1/2 **(E)**, p-AKT **(F)**, and p-STAT3 **(G)** in the pancreas were assayed. Graphs show means ± sem, ^*^P < 0.05, ^**^P < 0.01 (*n* = 5 mice/treatment, unpaired *t*-test).

To identify whether RAGE or TLR9 is required for PDAC development in KCH mice, we crossed KCH mice with *Rage*^−/−^ or *Tlr9*^−/−^ mice to generate KCHR (*Pdx1-Cre;K-Ras^G12D/+^*;*Hmgb1*^−/−^;*Rage*^−/−^) and KCHT9 (*Pdx1-Cre;K-Ras^G12D/+^*;*Hmgb1*^−/−^;*Tlr9*^−/−^) mice. Notably, deletion of RAGE (but not TLR9) significantly prolonged animal survival (Figure 4A), decreased formation of pancreatic lesions (Figure 4B), increased normal acinar structures (Figure 4B), limited tumor metastasis/invasion to the liver, lung, and kidney (Figure 4C), reduced serum IL-6 levels (Figure 4D), and diminished pancreatic levels of p-ERK1/2 (Figure 4E), p-AKT (Figure 4F) and p-STAT3 (Figure 4G) in KCHR mice compared with KCH mice. These findings suggest that RAGE (but not TLR9) is required for oncogenic K-Ras activation in KCH tumor development.

Moreover, KCHR mice (but not KCHT9 mice) exhibited decreased pancreatic expression of SOX9 (an ADM marker), vimentin (a stromal marker), Ki-67 (a proliferation marker), and MMP7 (an invasion marker) compared with KCH mice (Supplementary information, Figure S11). We extended our findings with cultured peritoneal macrophages and PDAC cells from KCH, KCHR, and KCHT9 mice. We found that loss of RAGE (but not TLR9) diminished recombinant nucleosome-induced IL-6 release in isolated peritoneal macrophages and PDAC cells *in vitro* (Supplementary information, Figure S12). Together, these data suggest that RAGE is required for K-Ras-driven tumorigenesis and development partly through mediating extracellular nucleosome activity in KCH mice.

### Pharmacologic inhibition of nuclear HMGB1 loss and release limits K-Ras-induced pancreatic lesions

**Figure 5:**
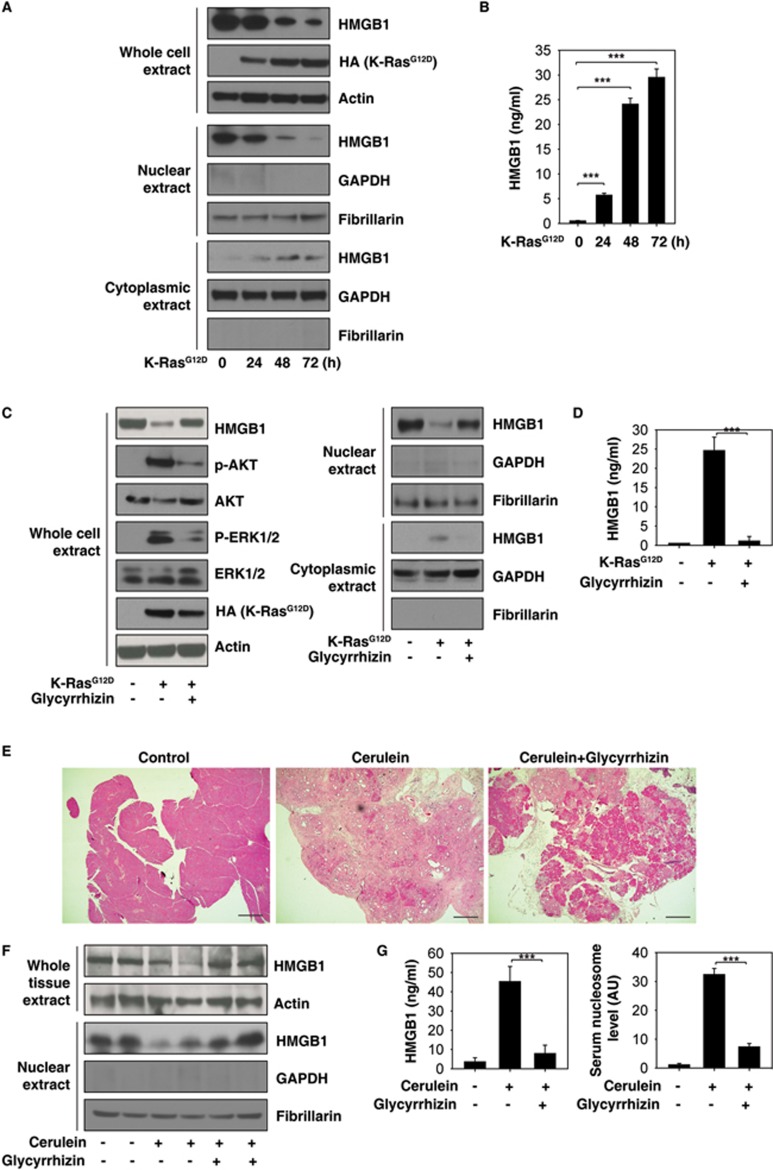
Inhibition of nuclear HMGB1 loss and release by glycyrrhizin limits K-Ras-induced PanIN formation. **(A**, **B)** Effects of *K-Ras*^G12D^ transfection on HMGB1 expression **(A)** and release **(B)** in primary mouse pancreatic acinar cells (*n* = 3, ^***^P < 0.001, data are expressed as means ± sem, unpaired *t*-test). **(C**, **D)** Effects of glycyrrhizin (500 μM) on HMGB1 expression **(C)** and release **(D)** in primary mouse pancreatic acinar cells after K-Ras^G12D^ transfection for 48 h (*n* = 3, ^***^P < 0.001, data are expressed as means ± sem, unpaired *t*-test). **(E-G)** Effects of glycyrrhizin (10 mg/kg) on PanIN formation **(E)** and HMGB1 expression **(F)** and release **(G)** on day 21 in a cerulein-mediated accelerated oncogenic *K-Ras* mouse model of pancreatic cancer (*n* = 5 mice/treatment, ^***^P < 0.001, data are expressed as means ± sem, unpaired *t*-test).

We next investigated the expression and release of HMGB1 in K-Ras^G12D^-induced oncogenic transformation. Intracellular HMGB1 (especially nuclear HMGB1) expression was progressively reduced over 72h (Figure 5A), whereas extracellular HMGB1 progressively increased over the same interval (Figure 5B) in response to K-Ras^G12D^-induced oncogenic transformation of normal pancreatic acinar cells. This suggests that HMGB1 loss and release is an important event in pancreatic tumorigenesis. Several compounds have been identified that directly or indirectly attenuate the translocation and release of HMGB1. Among these compounds, glycyrrhizin is a direct HMGB1 inhibitor with anti-inflammatory and antioxidant activity^46,47^. We therefore examined the effect of pharmacological inhibition of HMGB1 by glycyrrhizin in K-Ras^G12D^-induced oncogenic transformation. We found that glycyrrhizin treatment led to a remarkable suppression of nuclear loss (Figure 5C) and extracellular release of HMGB1 (Figure 5D) in K-Ras^G12D^-induced oncogenic transformation. Moreover, glycyrrhizin also blocked K-Ras^G12D^-induced increase in p-AKT and p-ERK1/2 (Figure 5C), suggesting that pharmacologic inhibition of nuclear HMGB1 loss and release can limit activation of K-Ras signaling.

It is well established that cerulein treatment-induced pancreatitis accelerates PanIN formation and stromal response in KC mice^48,49^. Next, we determined whether pharmacologic inhibition of nuclear HMGB1 loss and release by glycyrrhizin prevents K-Ras-induced pancreatic cancer initiation under inflammatory conditions. Indeed, we found that glycyrrhizin treatment prevented PanIN development preserving normal acinar structures (Figure 5E), sustaining nuclear HMGB1 expression (Figure 5F), and decreasing circulating HMGB1 and nucleosome levels (Figure 5G) in the cerulein-mediated accelerated oncogenic K-Ras mouse model of pancreatic cancer. Hence, prevention of loss of nuclear HMGB1 and subsequent nuclear DAMP (e.g., nucleosome and HMGB1) release may reveal a new PDAC prevention strategy.

### Reduced HMGB1 expression correlates with poor survival in pancreatic cancer patients

**Figure 6:**
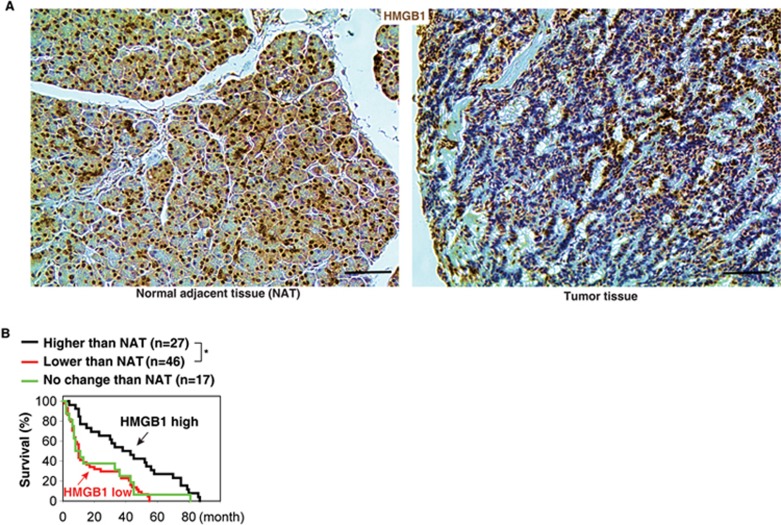
Reduced HMGB1 expression in pancreatic tissue compared with normal adjacent tissue (NAT) in pancreatic cancer patients **(A)** with poor survival outcomes **(B)** (^*^P < 0.05, log-rank test).

To determine whether HMGB1 expression is aberrant in human pancreatic cancer, we used pancreas tissue microarrays to analyze a cohort of 90 patients with pancreatic cancer. The expression of HMGB1 protein, especially nuclear HMGB1, was decreased in pancreatic tumor compared with adjacent normal pancreatic tissue and normal tissue (Figure 6A). Furthermore, the proportion of PDAC tumors that had lower HMGB1 expression than normal adjacent tissue (low HMGB1 group) correlated with a worse outcome (Figure 6B). The median survival time of the low HMGB1 group versus the high group was 10 months versus 43 months. However, HMGB1 mutation was not observed in pancreatic cancer patients according to the cBioPortal website (http://www.cbioportal.org). These findings indicated that loss of HMGB1 expression (but not HMGB1 mutation) may be a pathological event in human PDAC progression.

## Discussion

**Figure 7:**
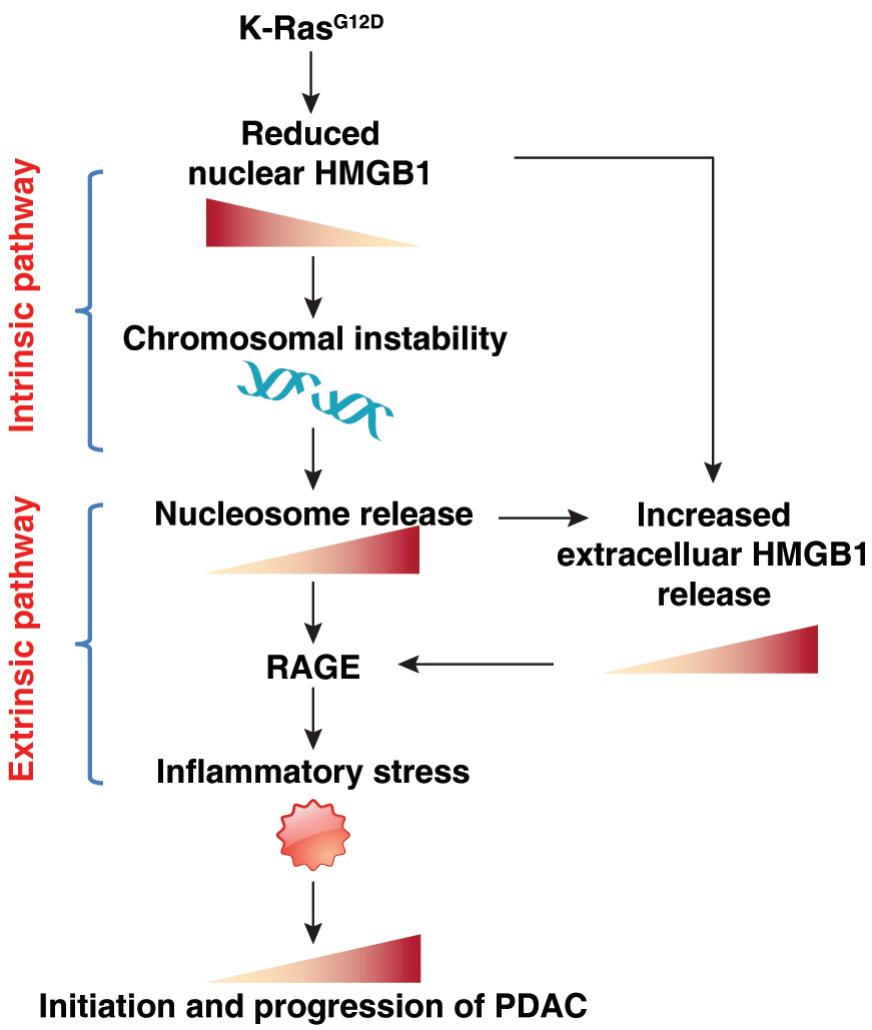
Schematic depicting HMGB1 deficiency-mediated nucleosome release linking chromosomal instability to the inflammatory response in K-Ras-driven PDAC. Oncogenic K-Ras^G12D^ leads to HMGB1 translocation and release into the extracellular space. Loss of intracellular HMGB1 increases chromosomal instability and promotes nucleosome release (current study). In addition, we previously demonstrated that extracellular nucleosome activates innate immune cells (e.g., macrophages) to secrete HMGB1 into circulation^24^. Both extracellular nucleosome and HMGB1 exacerbates PDAC development by enhancing RAGE-dependent inflammatory responses.

The pathogenesis of PDAC is partly attributable to intrinsic chromosome instability and extrinsic inflammation activation^25,50,51^. However, the molecular link between these two events in pancreatic tumorigenesis has not yet been fully established. We show that intracellular HMGB1 confers remarkable suppression of oncogenic K-Ras-driven pancreatic tumorigenesis by inhibiting pro-inflammatory nucleosome release mediated by chromosome instability characterized by chromosome rearrangements and telomeric defects (Figure 7). Conditional genetic ablation of either single or both alleles of HMGB1 in the pancreas renders mice extremely sensitive to oncogenic K-Ras-driven initiation of precursor lesions at birth, as well as tumor metastasis/invasion at six weeks. Loss of HMGB1 in the pancreas enables oxidative DNA damage and emergent chromosomal instability, which leads to inflammatory nucleosome release and propagates K-Ras-driven pancreatic tumorigenesis. Extracellular nucleosomes promote IL-6 secretion by infiltrating macrophages/neutrophils and sustain oncogenic K-Ras signaling activation in pancreatic lesions. Neutralizing antibodies to IL-6 or histone H3 or knockout of RAGE all limit K-Ras signaling activation, prevent cancer development and metastasis/invasion, and prolong animal survival in KCH mice. Pharmacological inhibition of intracellular HMGB1 loss limits K-Ras-driven tumorigenesis in mice. This evidence strongly indicates that intracellular HMGB1 is a tumor suppressor of PDAC.

HMGB1 was initially defined in 1973 as a non-histone chromatin protein. Inside the nucleus, HMGB1 interacts with DNA and histones to determine chromatin structure^32^ and regulates key processes such as transcription^52^. HMGB1 has other intracellular actions; when it translocates to the cytoplasm, it can mediate autophagy^53,54^. Outside the cell, HMGB1 acquires a new identity to serve as a danger signal or a DAMP^55^. Elevated serum HMGB1 levels are implicated in disease with sterile inflammation and infection^19^. Elevated serum HMGB1 levels also correlate with the presence, progression, and prognosis of PDAC^56^. Although serum levels are increased in PDAC patients, the expression of intracellular HMGB1 in pancreatic tissue remains unclear. Our tissue microarray analysis of patients with pancreatic cancer reveals a significant correlation between reduced HMGB1 expression and poor survival. We also observed that K-Ras^G12D^-induced oncogenic transformation led to HMGB1 translocation and release into the extracellular space. On one hand, loss of intracellular HMGB1 increases chromosomal instability and telomere attrition, whereas increased extracellular nuclear DAMPs (e.g., nucleosome) in HMGB1-deficient cancer cells cause an excessive inflammation response in the tumor microenvironment. These HMGB1-mediated pathogenic changes drive the initiation and development of PDAC.

Telomeres are the protective DNA-protein complexes found at the end of eukaryotic chromosomes, whereas subtelomeres are segments of DNA between telomeric caps and chromatin^57^. Previous studies showed that knockout of the HMGB1 in mouse embryonic fibroblasts or knockdown of HMGB1 in human breast cancer cell lines resulted in a decline in telomerase activity and telomere dysfunction^58,59^. We found here that loss of HMGB1 leads to abnormal telomeres and nucleosome release in PDAC cells. It will be interesting to uncover whether HMGB1-mediated release of nucleosome is subtelomere- or telomere-specific.

Metaplasia is defined by the conversion or replacement of one differentiated cell type with another in the context of a given tissue. In some tissues, metaplasia is associated with an increased risk of cancer^60^. Investigation of metaplasia might also provide clues to the cell of cancer origin. To that end, pancreatic acinar cells have the capacity to undergo metaplasia to a ductal cell phenotype in the origin of PDAC^61,62,63,64^. ADM might represent reprogramming of a progenitor population, direct transdifferentiation of acinar cells to ductal cells, or transdifferentiation via an intermediate cell type (potentially a progenitor cell)^65^. We demonstrate that loss of HMGB1 increases *K-Ras*-driven ADM in pancreatic tumorigenesis. The molecular basis underlying the development of ADM involves a number of seemingly diverse pathways and molecules, including transcriptional factor^65,66,67,68^. Sox9 is a critical transcriptional factor of the HMG box family, which not only sustains proliferation and survival of the pancreatic progenitor cell pool^69,70^, but also triggers ADM in neoplastic transformation including PanINs, IPMNs, and MCNs^28,63,71,72,73^. We observed that loss of HMGB1 increased PanINs, IPMNs, and MCNs, which is associated with increased Sox9 expression in K-Ras-driven pancreatic tumorigenesis. The detailed molecular basis for HMGB1 function in the formation of ADM and mixed precursor lesions warrants further investigation.

Growing evidence shows that tumors, including PDAC, are sustained and promoted by inflammatory signals from the surrounding microenvironment. Our current study identifies a role of nucleosome release in the pancreatic tumor microenvironment. Besides exerting intranuclear functions, nucleosomes act as inflammatory DAMPs when they are released into the extracellular space in oxidative injury and genomic instability^74^. Indeed, increased serum levels of nucleosomes, including the component histones and DNA, have been observed in many cancers and correlate with disease progression^75,76^. As nucleosomes are stable structures in the circulation^77^, they could be a valuable source of biomarkers in PDAC. Our current study provides evidence that extracellular nucleosomes promote IL-6 secretion by myeloid cells, which in turn sustains STAT3 and K-Ras signaling in pancreatic cancer cells. These findings may represent a new response of the inflammatory microenvironment in tumor progression.

Both the present and previous studies highlight the important role of RAGE in the initiation of PDAC. RAGE is a transmembrane receptor of the immunoglobulin gene superfamily and a multifunctional receptor within the tumor microenvironment^20^. RAGE and its ligands are linked to the development and progression of several cancers by facilitating the maintenance of a chronic inflammatory state^78^ and/or by promotion of metastases^22^. We previously demonstrated that: (1) RAGE was highly expressed in mouse and human PDAC^39^; (2) Targeted genetic ablation of RAGE in mice prevented experimental acute pancreatitis^79^ and pancreatic cancer growth in the KC model^39^; and (3) RAGE was essential for pancreatitis^79^ and oncogenic K-Ras-mediated hypoxic signaling in pancreatic cancer development^80^. Several studies have found that RAGE is a nucleic acid receptor for recognition of various DNA and RNA molecules^42,81^. Our recent study shows that RAGE is a potential receptor for nuclear DAMP complex (HMGB1-histone-DNA) in macrophages^82^. Nucleosomes may signal through receptors such as TLRs, as well as other yet unidentified receptors, depending on cell type^36^. In the present study, we further identified RAGE (but not TLR9) as a receptor mediating extracellular nucleosome activity in K-Ras-driven tumorigenesis in KCH mice. Pharmacological or genetic blocking of the nucleosome-RAGE pathway limits STAT3 and K-Ras signaling in KCH mice.

In summary, conditional knockout of HMGB1 in the pancreas leads to significant acceleration of K-Ras-driven carcinogenesis. Compared with other reported genetically engineered mouse models, the KCH model is extremely sensitive to oncogenic K-Ras signaling, with development of mixed precursor lesions at birth. HMGB1 translocation from the nucleus or deficiency-mediated nucleosome release can now be appreciated as key events linking chromosomal instability and the inflammatory response (Figure 7). This process requires activation of RAGE, a DAMP receptor that promotes inflammatory responses to HMGB1^83^, DNA^42^, and histones^79^. Thus, an impaired HMGB1-nucleosome-RAGE-mediated DAMP pathway contributes to K-Ras signaling activation and may represent a potent therapeutic target in PDAC. The KCH model provides an interesting and provocative model for future translational research and drug discovery.

## Materials and Methods

### Animals and *in vivo* models

We conducted all animal care and experiments in accordance with the Association for Assessment and Accreditation of Laboratory Animal Care guidelines (http://www.aaalac.org) and with approval from the University of Pittsburgh Institutional Animal Care and Use Committee. C57BL/6 mice were purchased from Jackson Laboratories.

*Hmgb1^flox/flox^* mice on C57BL/6 background were obtained from Dr Eugene B Chang (University of Chicago)^24,84^. The HMGB1 conditional knockout mice were genotyped with primers 5′-TGATGCGAACACGGCGTGCTCTA-3′ and 5′- GCACAAAGAATGCATATGAGGAC-3′. PCR conditions used were as follows: 94 °C for two minutes; then 30 cycles of 94 °C for 15 s, 62 °C for 30 s and 72 °C for 30 s; and then held at 72 °C for 5 min before cooling to 4 °C. WT mice were identified by a single band at 635 bp. HMGB1 floxed mice were identified by a single band at 700 bp.

*Rage*^−/−^ mice on C57BL/6 background were a kind gift from the late Dr Angelika Bierhaus (University of Heidelberg)^85^. Dr Bierhaus passed away on 15 April 2012 after a long and courageous battle with cancer. The RAGE knockout mice were genotyped with primers 5-CCTGGGTGCTGGTTCTTG-3′ and 5′- CTGAGGTCCGTGGCTAGG-3′. PCR conditions used were as follows: 94 °C for five minutes; then 30 cycles of 94 °C for 30 s, 60 °C for 30 s and 72 °C for 3 min; and then held at 72 °C for 5 minutes before cooling to 4 °C. *Rage*^+/+^ mice were identified by a single band at 1 750 bp. *Rage*^−/−^ mice were identified by a single band at 2 100 bp.

*Tlr9*^−/−^ mice lacked CpG motif on C57BL/6 background was obtained from Dr Timothy R Billiar (University of Pittsburgh)^86^. The *Tlr9*^−/−^ mice had a one base-pair mutation on CpG. The section of DNA for sequencing was ACGCCCCCAGT. The CCCCC 5 C's were a *Tlr9*^−/−^ and CCTCC were a *Tlr9*^+/+^.

*Pdx-1-Cre* and *K-ras^G12D/+^* transgenic mice on C57BL/6 background were received from the MMHCC/NCI Mouse Repository. These mice were crossed to generate indicated KC, KCH, KCHR, and KCHT9 animals. All mice were housed under a 12-h light-dark diurnal cycle with controlled temperature (21 °C to 23 °C) and provided with a standard rodent diet and water *ad libitum* throughout all experiments.

To study the effects of oxidative DNA damage on pancreatic tumorigenesis, four-week-old KCH mice were injected intraperitoneally (i.p.) with NAC (10 mg/kg, Sigma) five times per week for four weeks. To study the effects of extracellular nucleosome on pancreatic tumorigenesis, four-week-old KCH mice were injected i.p. with neutralizing anti-H3 (10 mg/kg, Abcam) or anti-IL-6 antibody (10 mg/kg, BioLegend) twice per week for 4 weeks.

To study the effects of HMGB1 inhibitor on pancreatitis-induced pancreatic tumorigenesis, four-week-old KC mice were treated with two sets of six hourly i.p. cerulein injections (50 μg/kg, Sigma) on alternating days separated by 24 h. These KC mice were then injected i.p. with glycyrrhizin (10 mg/kg, Sigma) five times per week for 2 weeks. The final day of cerulein injection was considered day 0.

### ELISA analysis

The amylase (Abcam), trypsin (Abcam), IL-6 (BioLegend), insulin (Thermo Fisher Scientific Inc.), and HMGB1 (Shino-Test Corporation) levels in serum or supernatant fractions from pancreatic homogenate were measured using ELISA according to the manufacturer's protocol. For measurement of serum nucleosomes, Cell Death Detection ELISA^plus^ (Roche Diagnostics) was used. It is based on a quantitative sandwich enzyme immunoassay principle. Monoclonal mouse antibodies directed against DNA (single-stranded and double-stranded) and histones (H1, H2A, H2B, H3, and H4) specifically detect mono- and oligonucleosomes. The anti-histone antibody additionally binds to the microtiter plate, whereas the anti-DNA antibody labeled with peroxidase reacts with 2,2′-azinobis(3-ethylbenzothiazoline sulfonate)^87^. Recombinant nucleosomes were purchased from EpiCypher.

### Image analysis

For histological analysis, pancreatic specimens were fixed with 10% buffered formalin, dehydrated in ethanol, embedded with paraffin, and stained with hematoxylin and eosin, Alcian blue, or Masson's trichrome. The fraction of preserved acinar area was calculated as previously described^88^. Pancreatic ductal dysplasia was graded according to established criteria^89^. Histological images were acquired using an EVOS FL Auto Cell Imaging System (Thermo Fisher Scientific, Inc.).

Immunofluorescent staining of mouse tissues was performed using antibodies directed against 8-OHDG (Abcam), γ-H2AX (Cell Signal Technology), p-ERK1/2 (Cell Signal Technology), p-AKT (Cell Signal Technology), p-STAT3 (Cell Signal Technology), amylase (Santa Cruz Biotechnology), CK19 (Developmental Studies Hybridoma Bank), SOX9 (Millipore), vimentin (Cell Signal Technology), ki67 (Abcam), MMP7 (Cell Signal Technology), RAGE (Santa Cruz Biotechnology), TLR2 (Santa Cruz Biotechnology), TLR4 (Abcam), TLR9 (Abcam), insulin (Cell Signal Technology), glucagon (Abcam), and DAPI (Invitrogen). Immunofluorescent images were acquired using an AxioObserver Z1 Microscope with Apotome (Carl Zeiss). Quantifications of images were performed by assessing 20X high-power fields per slide and 20 fields per mouse (five mice/group) in a blinded manner.

Immunohistochemistry in human pancreatic cancer tissue array was performed using antibodies directed against HMGB1 (Abcam). This tissue microarray (#HPan-Ade180Sur-01) was purchased from US Biomax and included 90 cases of pancreatic cancer tumor (one core/case) and matched normal adjacent tissue (one core/case). Clinical stage, survival information, smoking history, drinking history, diabetes history, hepatitis B information, and family medical history were available from the US Biomax website (http://www.biomax.us/tissue-arrays/Pancreas/HPan-Ade180Sur-01).

### Quantitative real-time PCR

Total RNA was extracted using TRI reagent (Sigma) according to the manufacturer's instructions. First-strand cDNA was synthesized from 1 μg of RNA using the iScript cDNA Synthesis kit (Bio-Rad, Hercules, CA). cDNA from various cell samples were amplified using real-time quantitative PCR with specific primers (*Rage*: 5′-CTGCGTGTTCTACGAGATTGCC-3′ and 5′-GACAAGGTGTGCCGATGACATC-3′ *Tlr2*: 5′- ACAGCAAGGTCTTCCTGGTTCC-3′ and 5′- GCTCCCTTACAGGCTGAGTTCT-3′ *TLR4*: 5′- AGCTTCTCCAATTTTTCAGAACTTC-3′ and 5′-TGAGAGGTGGTGTAAGCCATGC-3′ *Tlr9*: 5′- GCTGTCAATGGCTCTCAGTTCC-3′ and 5′- CCTGCAACTGTGGTAGCTCACT-3′) and the data were normalized to *Rn18s* ribosomal RNA (5′-CTTAGAGGGACAAGTGGCG-3′ and 5′-ACGCTGAGCCAGTCAGTGTA-3′). RT² profiler PCR array for telomeres or DNA damage signaling pathway was measured using a Kit from QIAGEN according to the manufacturer's protocol.

### *K-Ras*^G12D^ transfection

*K-Ras*^G12D^ plasmid (Addgene) was electroporated into primary mouse acinar cells using the Neon Transfection System (Life technologies) according to the manufacturer's protocol.

### Measuring glucose

Glucose concentration measurements were obtained from whole-blood samples using hand-held whole-blood glucose monitors (Bayer) according to the manufacturer's protocol.

### Western blot analysis

Western blot was used to analyze protein expression as described previously^54^. In brief, after extraction, proteins in cell lysates were first resolved by SDS-PAGE electrophoresis and then transferred to nitrocellulose membrane and subsequently incubated with primary antibodies (anti-HMGB1 (Novus), anti-actin (Sigma), anti-GAPDH (Sigma), anti-fibrillarin (Abcam), anti-AKT (Cell Signal Technology), anti-p-AKT (Cell Signal Technology), anti-ERK1/2 (Cell Signal Technology), and anti-p-ERK1/2 (Cell Signal Technology)). After incubation with peroxidase-conjugated secondary antibodies, the signals were visualized with enhanced chemiluminescence (Pierce, Rockford, IL, USA) according to the manufacturer's instructions.

### Statistical analysis

Data are presented as mean ± sem. Survival was measured using the Kaplan–Meier method. Statistical significance was determined with unpaired *t*-test or the log-rank test using SigmaPlot 11.0 software. *P* < 0.05 was considered significant.

## Author Contributions

RK and DT designed the research. RK, YX, QZ, WH, QJ, ZS, DZ, and DT performed the experiments. RK, HW, HJZ, MTL, and DT analyzed the results. JB, TRB, MTL, and HJZ provided important reagents. RK, HW, and DT wrote the paper. JB, TRB, DLB, MTL, and HJZ edited and commented on the manuscript.

## Competing Financial Interests

The authors declare no competing financial interests.

## Supplementary Material

Supplementary information, Figure S1 Generation of KCH mice.

Supplementary information, Figure S2 Histologic progression of pancreas shown by hematoxylin and eosin staining in KC, CH, KCH, and KCH^+/−^ mice.

Supplementary information, Figure S3 Representative hematoxylin and eosin stains of pancreatic intraepithelial neoplasms (PanINs) (a), mucinous cystic neoplasms (MCNs) (b), intraductal papillary mucinous neoplasms (IPMNs) (c), and pancreatic ductal adenocarcinoma (PDAC) (d) in KCH mice at six weeks of age.

Supplementary information, Figure S4 Loss of HMGB1 promotes pancreatic function insufficiency during K-Ras-driven tumor development.

Supplementary information, Figure S5 KCH and KCH^+/−^ mice exhibit significant acinar-to-ductal metaplasia (ADM).

Supplementary information, Figure S6 KCH and KCH^+/−^ mice exhibit significant duct-like lesions.

Supplementary information, Figure S7 KCH and KCH^+/−^ mice exhibit a significant stromal response.

Supplementary information, Figure S8 KCH and KCH^+/−^ mice exhibit significant proliferation and invasion.

Supplementary information, Figure S9 Pancreata from KCH mice exhibit abnormal mRNA expression of genes involved in regulating the DNA damage response (A) and telomere maintenance (B) by RT^2^ Profiler^™^ PCR Array (n=3 mice/genotype).

Supplementary information, Figure S10 KCH mice exhibit abnormal expression of nucleosome receptors.

Supplementary information, Figure S11 KCHR mice (but not KCHT9 mice) exhibited decreased expression of SOX9, vimentin, Ki-67, and MMP7 in pancreas compared with KCH mice at 12 weeks of age (n=5 mice/genotype, ^***^p < 0.001, data are expressed as means ± s.e.m, unpaired t-test).

Supplementary information, Figure S12 (A) Loss of RAGE (but not TLR9) diminished recombinant nucleosomes-induced IL-6 release in isolated peritoneal macrophages and PDAC cells from indicated mice at 6 weeks of age (n=3, ^***^p < 0.001, data are expressed as means ± s.e.m, unpaired t-test versus KCH group).

Supplementary information, Table S1 Data on tumor metastasis and invasion in KC, KCH, and KCH^+/−^ mice
