# Supplementary material for: Intracellular HMGB1 as a novel tumor suppressor of pancreatic cancer
Source: Cell Res. 2017 Apr 4;27(7):916–32. doi: 10.1038/cr.2017.51 (PMC5518983; doi:10.1038/cr.2017.51)
Supplement: Supplementary information, Figure S1 — Generation of KCH mice. [file cr201751x1.pdf]

## Supplementary Information

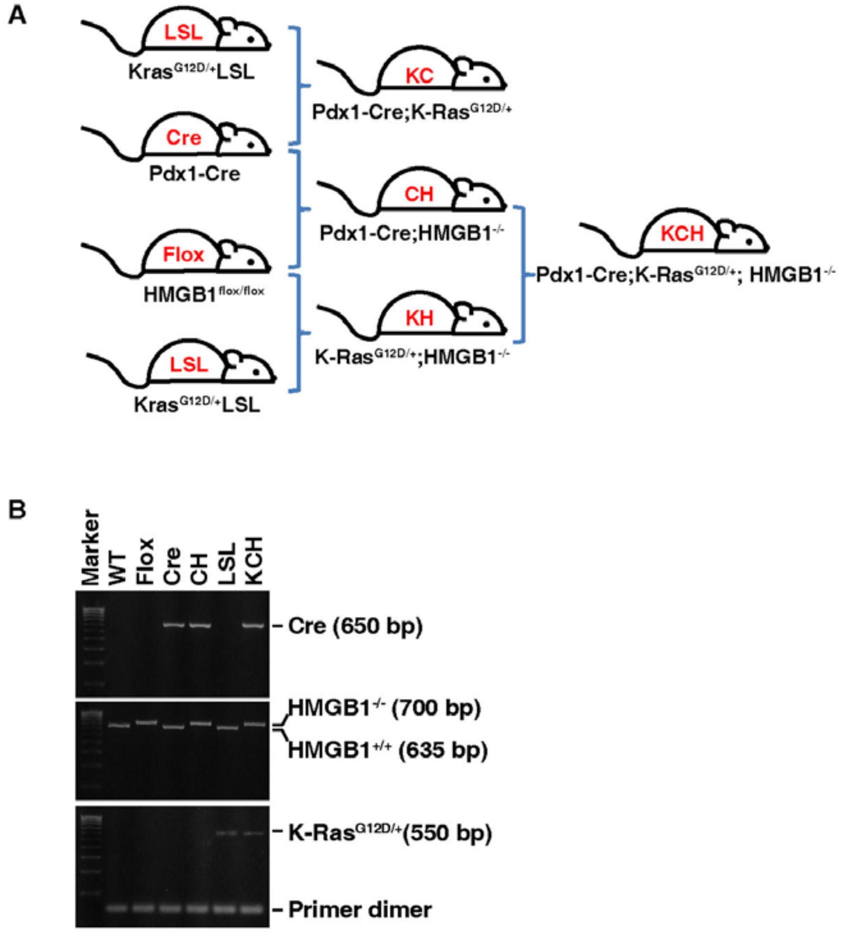


**Figure S1. Generation of KCH mice.** (A) Breeding scheme for producing transgenic mice. (B) Genotype identification of transgenic mice based on RT-PCR.
