# Supplementary material for: Intracellular HMGB1 as a novel tumor suppressor of pancreatic cancer
Source: Cell Res. 2017 Apr 4;27(7):916–32. doi: 10.1038/cr.2017.51 (PMC5518983; doi:10.1038/cr.2017.51)
Supplement: Supplementary information, Figure S2 — Histologic progression of pancreas shown by hematoxylin and eosin staining in KC, CH, KCH, and KCH+/− mice. [file cr201751x2.pdf]

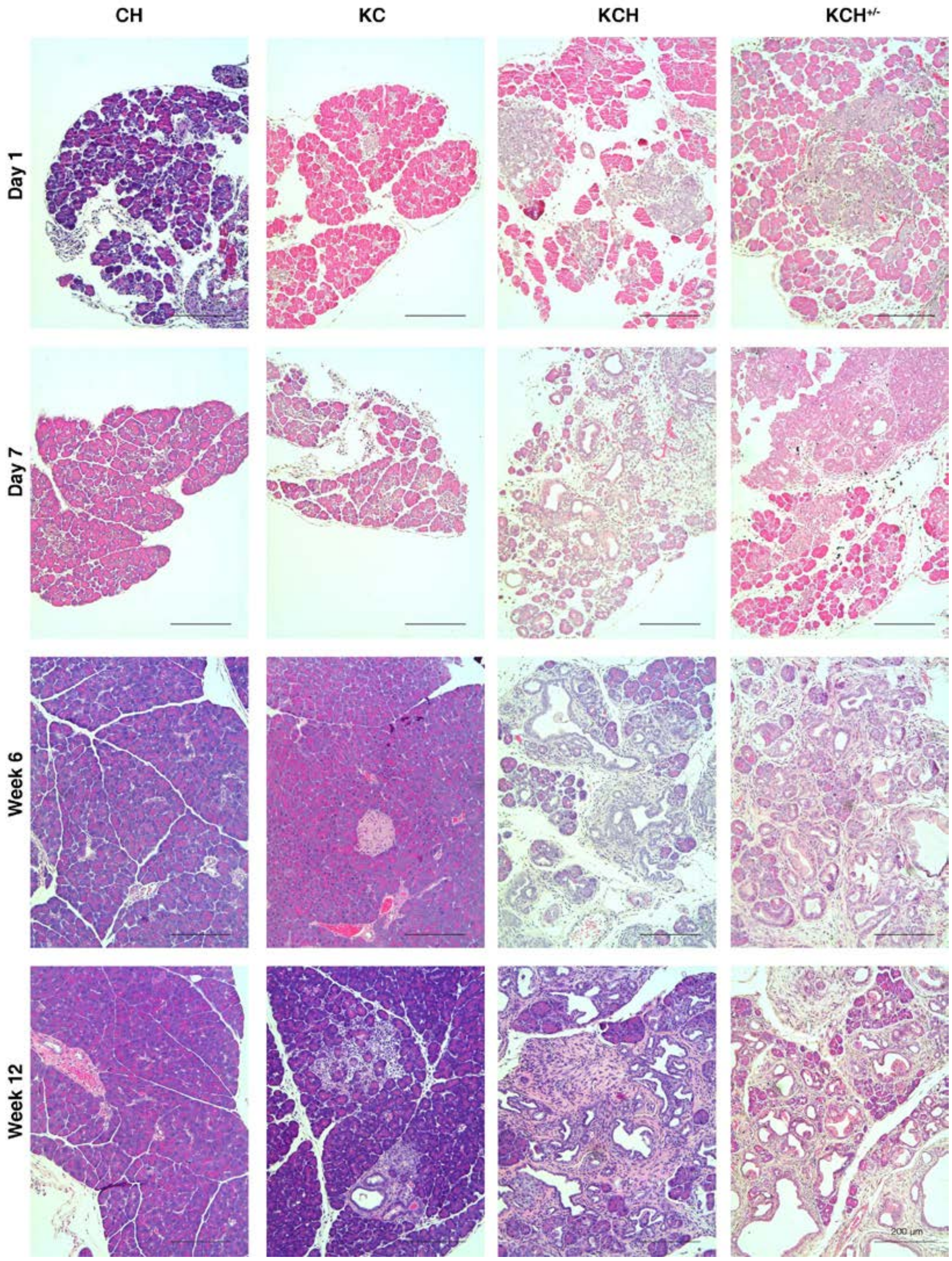

Figure S2. Histologic progression of pancreas shown by hematoxylin and eosin staining in KC, CH, KCH, and KCH$^{+/-}$ mice.
