# Supplementary material for: Intracellular HMGB1 as a novel tumor suppressor of pancreatic cancer
Source: Cell Res. 2017 Apr 4;27(7):916–32. doi: 10.1038/cr.2017.51 (PMC5518983; doi:10.1038/cr.2017.51)
Supplement: Supplementary information, Figure S3 — Representative hematoxylin and eosin stains of pancreatic intraepithelial neoplasms (PanINs) (a), mucinous cystic neoplasms (MCNs) (b), intraductal papillary mucinous neoplasms (IPMNs) (c), and pancreatic ductal adenocarcinoma (PDAC) (d) in KCH mice at six weeks of age. [file cr201751x3.pdf]

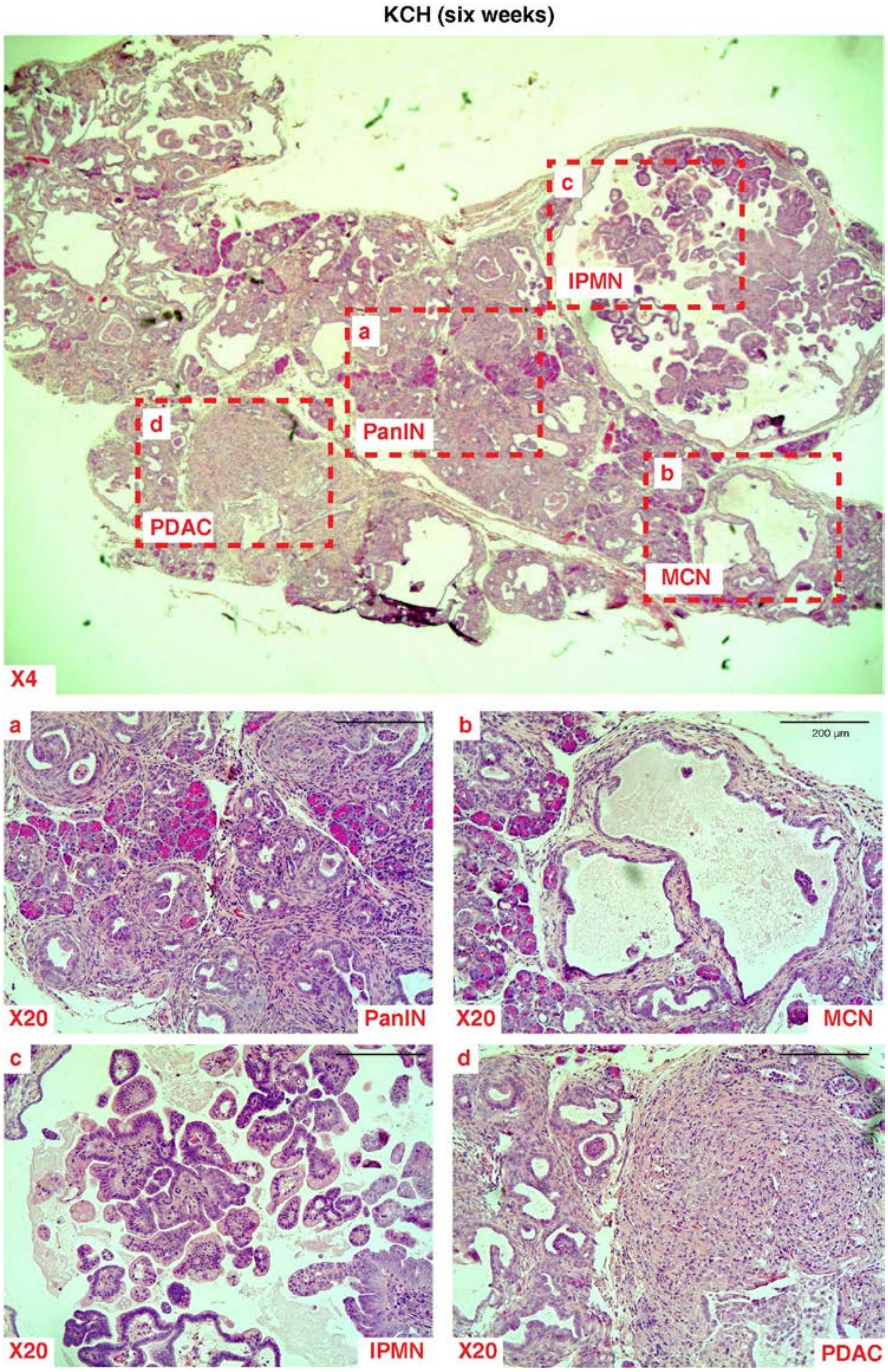

Figure S3. Representative hematoxylin and eosin stains of pancreatic intraepithelial neoplasms (PanINs) (a), mucinous cystic neoplasms (MCNs) (b), intraductal papillary mucinous neoplasms (IPMNs) (c), and pancreatic ductal adenocarcinoma (PDAC) (d) in KCH mice at six weeks of age.
