# Supplementary material for: Intracellular HMGB1 as a novel tumor suppressor of pancreatic cancer
Source: Cell Res. 2017 Apr 4;27(7):916–32. doi: 10.1038/cr.2017.51 (PMC5518983; doi:10.1038/cr.2017.51)
Supplement: Supplementary information, Figure S4 — Loss of HMGB1 promotes pancreatic function insufficiency during K-Ras-driven tumor development. [file cr201751x4.pdf]

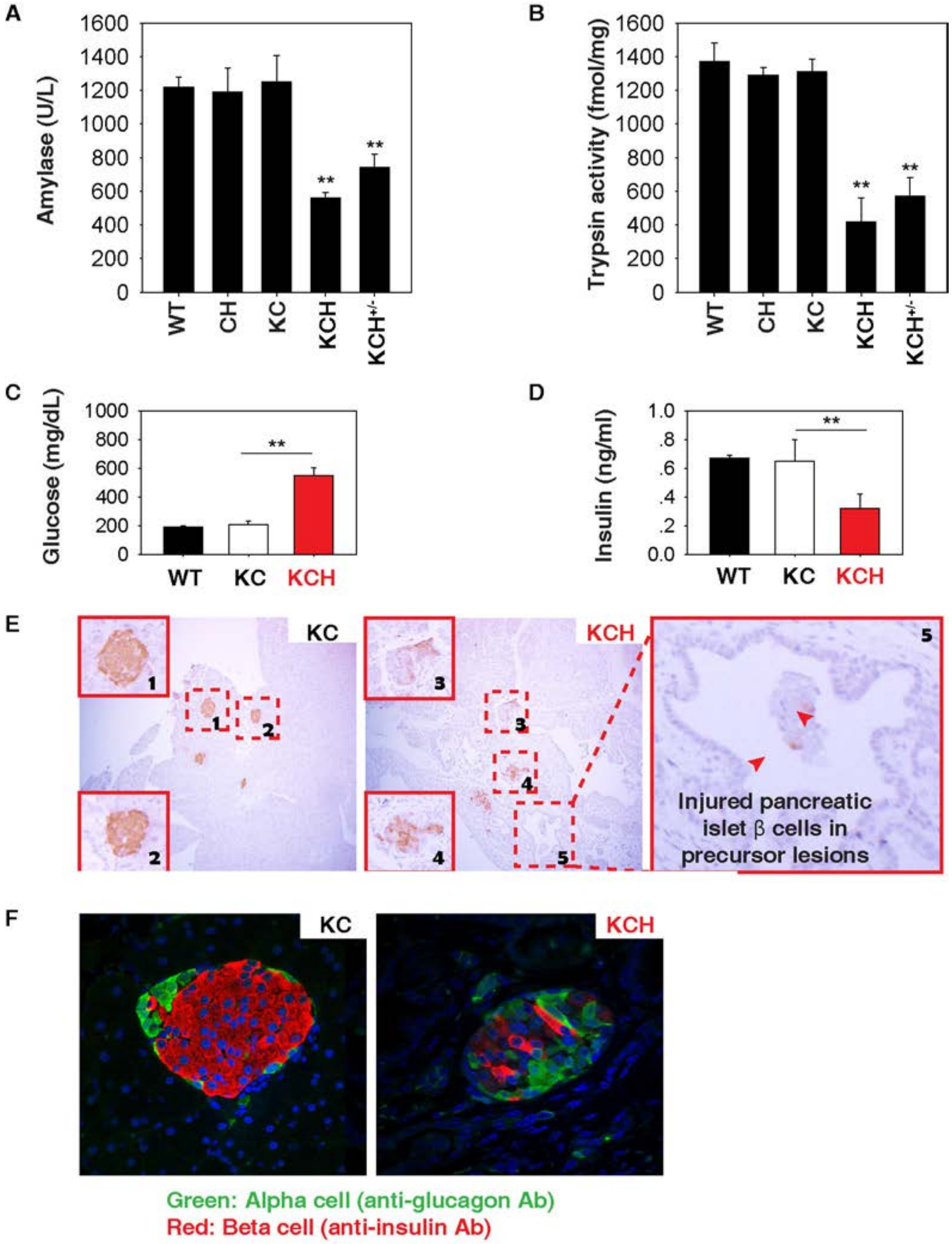

Figure S4. Loss of HMGB1 promotes pancreatic function insufficiency during K-Ras-driven tumor development. (A, B) Analysis of amylase levels in serum and trypsin activity in pancreata from indicated mice at six weeks of age (n=3 mice/genotype, **p < 0.01 versus KC group, data are expressed as means ± s.e.m, unpaired t-test). (C, D) Blood glucose (C) and serum insulin (D) levels in WT, KC, and KCH mice at six weeks (n=3 mice/genotype, **p < 0.01 versus KC group, data are expressed as means ± s.e.m, unpaired t-test). (E) Immunofluorescent staining of islet with insulin (brown) antibody in pancreatic tissue from KC and KCH mice at six weeks. (F) Immunohistochemical staining of islet alpha cell with glucagon antibody (green) and beta cell with insulin (red) antibody in pancreatic tissue from KC and KCH mice at six weeks.
