# Supplementary material for: Intracellular HMGB1 as a novel tumor suppressor of pancreatic cancer
Source: Cell Res. 2017 Apr 4;27(7):916–32. doi: 10.1038/cr.2017.51 (PMC5518983; doi:10.1038/cr.2017.51)
Supplement: Supplementary information, Figure S5 — KCH and KCH+/− mice exhibit significant acinar-to-ductal metaplasia (ADM). [file cr201751x5.pdf]

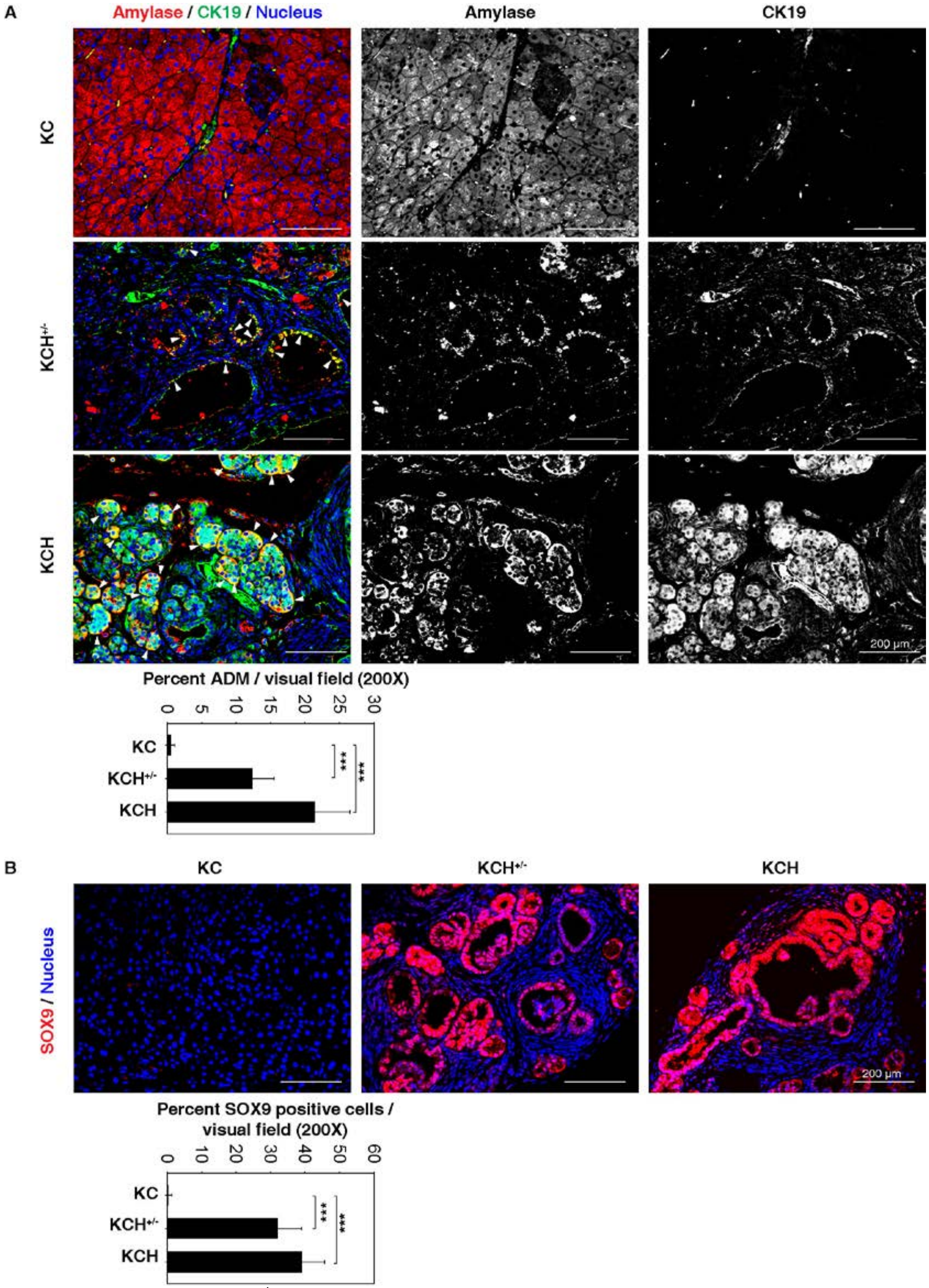

Figure S5. KCH and KCH$^{+/-}$ mice exhibit significant acinar-to-ductal metaplasia (ADM). (A) Analysis of ADM in pancreas by immunofluorescent (IF) staining of nuclei (blue), amylase (red), and CK19 (green) from indicated mice at six weeks of age (n=5 mice/genotype, ***p < 0.001, data are expressed as means ± s.e.m, unpaired t-test). (B) IF staining of nuclei (blue) and the ADM marker SOX9 (red) in pancreata from indicated mice at six weeks of age (n=5 mice/genotype, ***p < 0.001, data are expressed as means ± s.e.m, unpaired t-test).
