# Supplementary material for: Intracellular HMGB1 as a novel tumor suppressor of pancreatic cancer
Source: Cell Res. 2017 Apr 4;27(7):916–32. doi: 10.1038/cr.2017.51 (PMC5518983; doi:10.1038/cr.2017.51)
Supplement: Supplementary information, Figure S6 — KCH and KCH+/− mice exhibit significant duct-like lesions. [file cr201751x6.pdf]

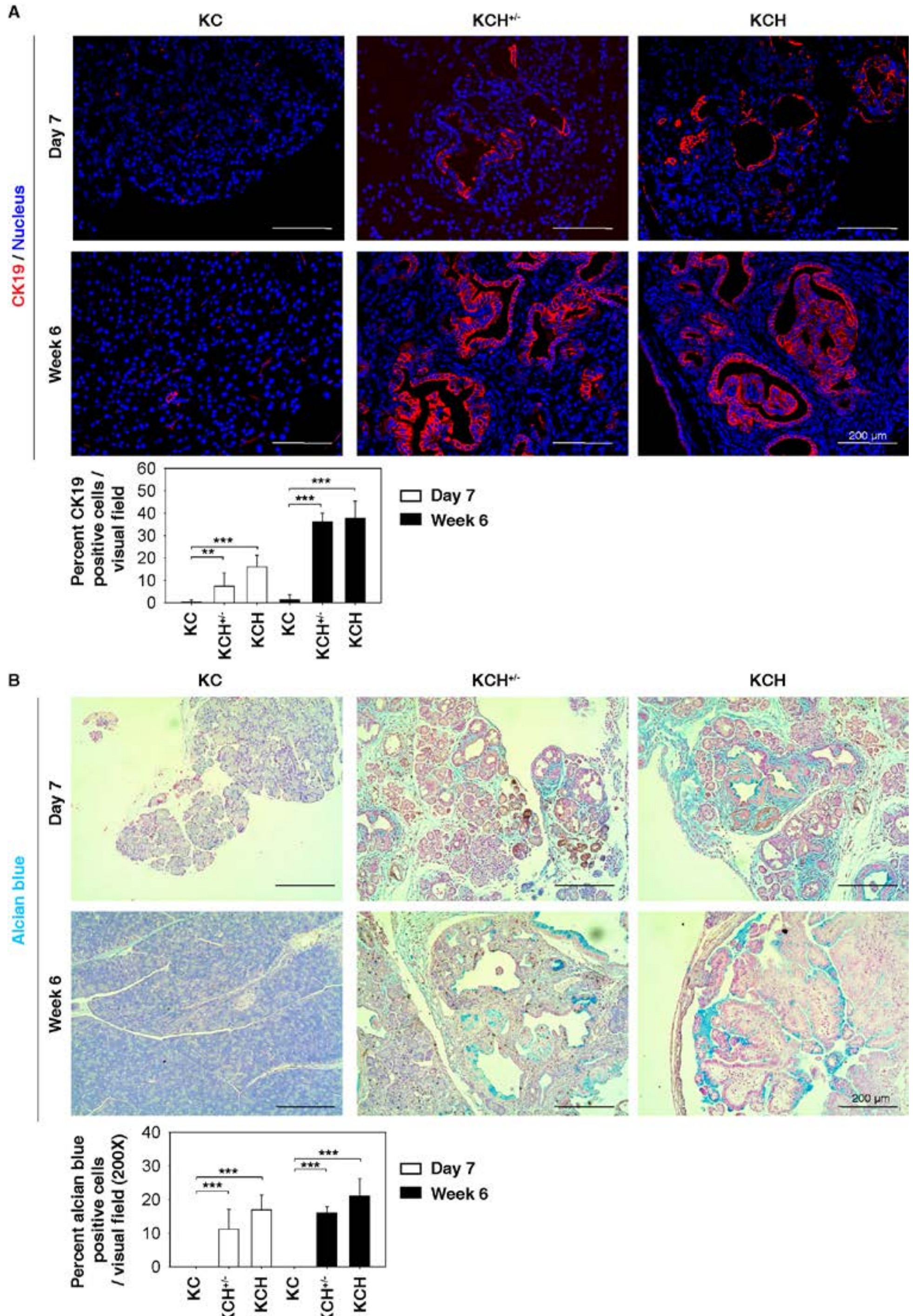

Figure S6. KCH and KCH$^{+/-}$ mice exhibit significant duct-like lesions. (A) Immunofluorescent staining of nuclei (blue) and CK19 (red) from indicated mice at six weeks of age (n=5 mice/genotype, **p < 0.01, ***p < 0.001, data are expressed as means ± s.e.m, unpaired t-test). (B) Mucin associated with ductal cells stained with Alcian blue in pancreata from indicated mice at six weeks of age (n=5 mice/genotype, ***p < 0.001, data are expressed as means ± s.e.m, unpaired t-test).
