# Supplementary material for: Intracellular HMGB1 as a novel tumor suppressor of pancreatic cancer
Source: Cell Res. 2017 Apr 4;27(7):916–32. doi: 10.1038/cr.2017.51 (PMC5518983; doi:10.1038/cr.2017.51)
Supplement: Supplementary information, Figure S9 — Pancreata from KCH mice exhibit abnormal mRNA expression of genes involved in regulating the DNA damage response (A) and telomere maintenance (B) by RT2 Profiler™ PCR Array (n=3 mice/genotype). [file cr201751x9.pdf]

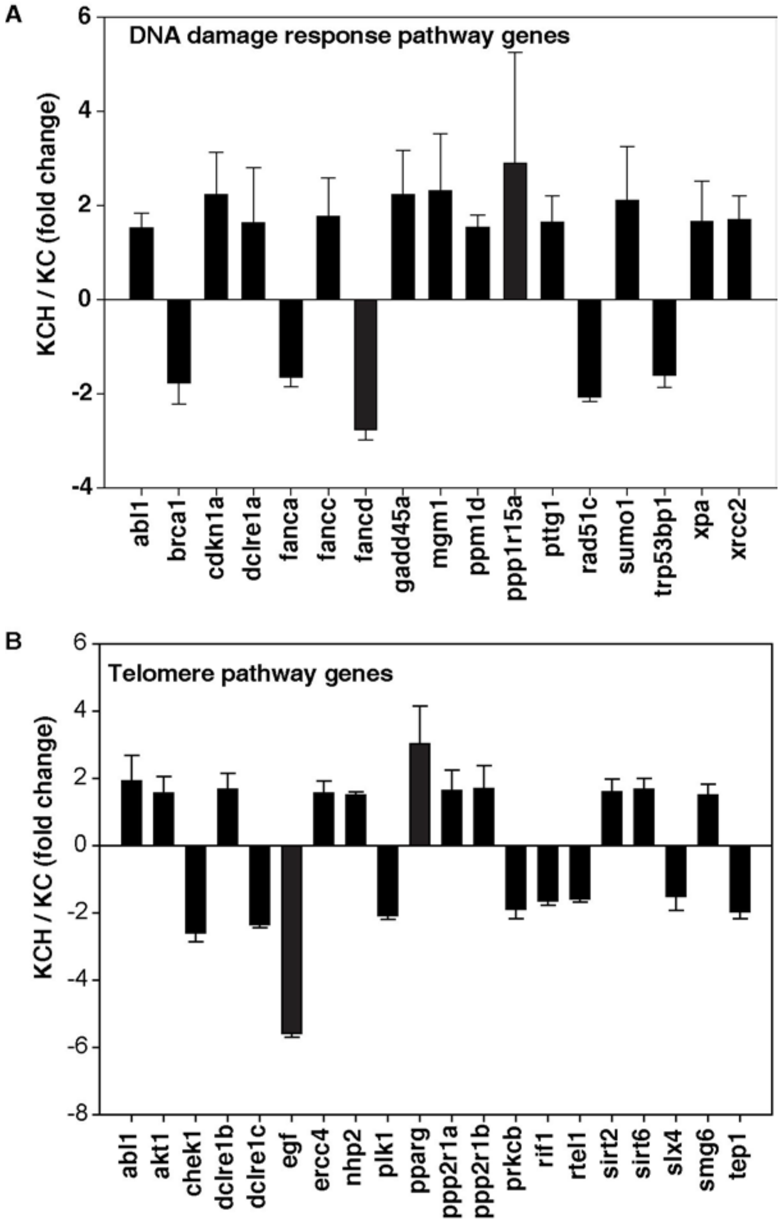

Figure S9. Pancreata from KCH mice exhibit abnormal mRNA expression of genes involved in regulating the DNA damage response (A) and telomere maintenance (B) by RT^2 Profiler™ PCR Array (n=3 mice/genotype).
