# Supplementary material for: Intracellular HMGB1 as a novel tumor suppressor of pancreatic cancer
Source: Cell Res. 2017 Apr 4;27(7):916–32. doi: 10.1038/cr.2017.51 (PMC5518983; doi:10.1038/cr.2017.51)
Supplement: Supplementary information, Figure S10 — KCH mice exhibit abnormal expression of nucleosome receptors. [file cr201751x10.pdf]

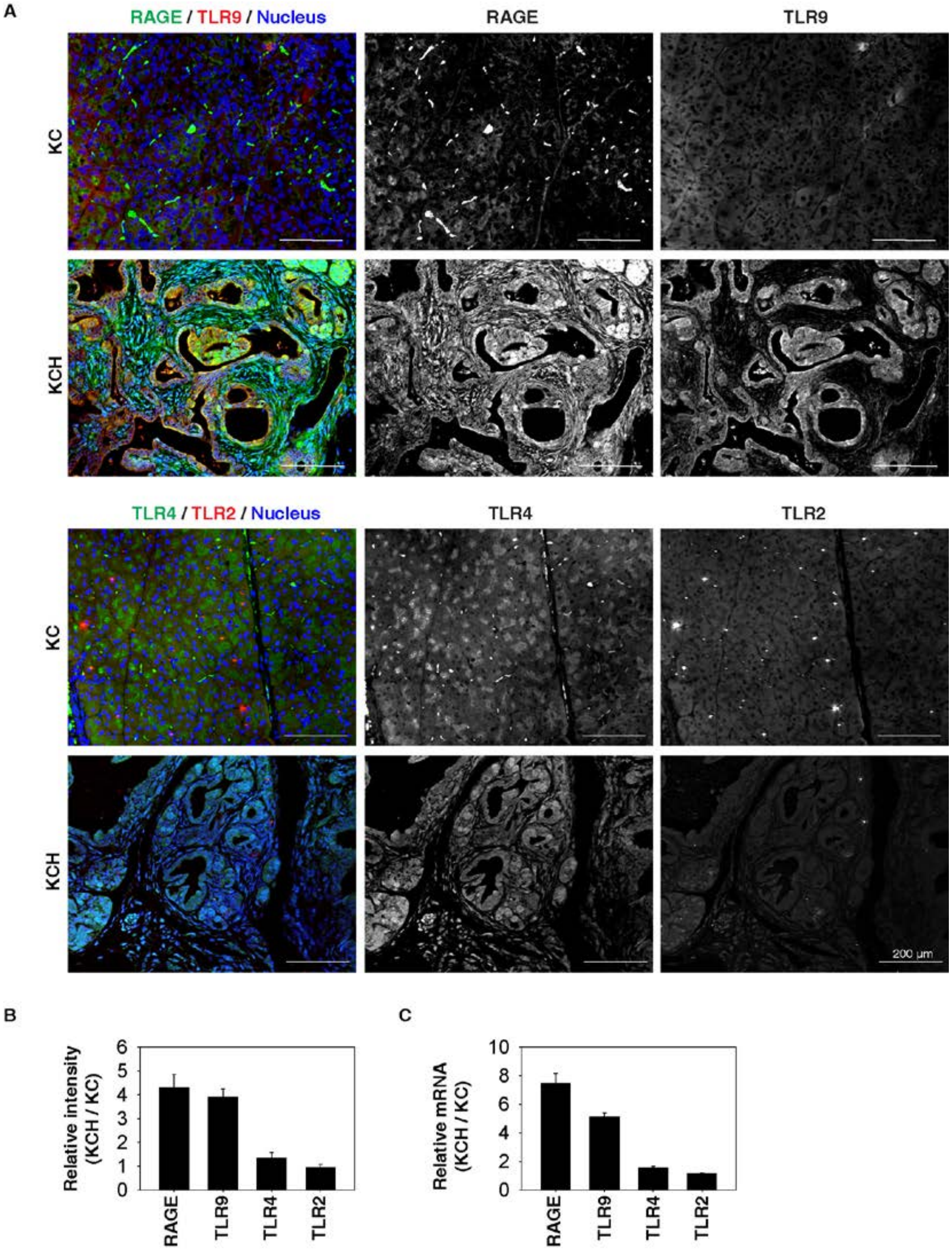

Figure S10. KCH mice exhibit abnormal expression of nucleosome receptors. (A-B) Immunofluorescent staining of nuclei (blue), RAGE (green), TLR9 (red), TLR4 (green), or TLR2 (red) from KC and KCH mice at six weeks of age (n=5 mice/genotype, data are expressed as means ± s.e.m). (C) Fold change of nucleosome receptor mRNA expression in KC and KCH mice by Q-PCR analysis (n=3 mice/genotype, data are expressed as means ± s.e.m).
