# Supplementary material for: Intracellular HMGB1 as a novel tumor suppressor of pancreatic cancer
Source: Cell Res. 2017 Apr 4;27(7):916–32. doi: 10.1038/cr.2017.51 (PMC5518983; doi:10.1038/cr.2017.51)
Supplement: Supplementary information, Figure S11 — KCHR mice (but not KCHT9 mice) exhibited decreased expression of SOX9, vimentin, Ki-67, and MMP7 in pancreas compared with KCH mice at 12 weeks of age (n=5 mice/genotype, ***p < 0.001, data are expressed as means ± s.e.m, unpaired t-test). [file cr201751x11.pdf]

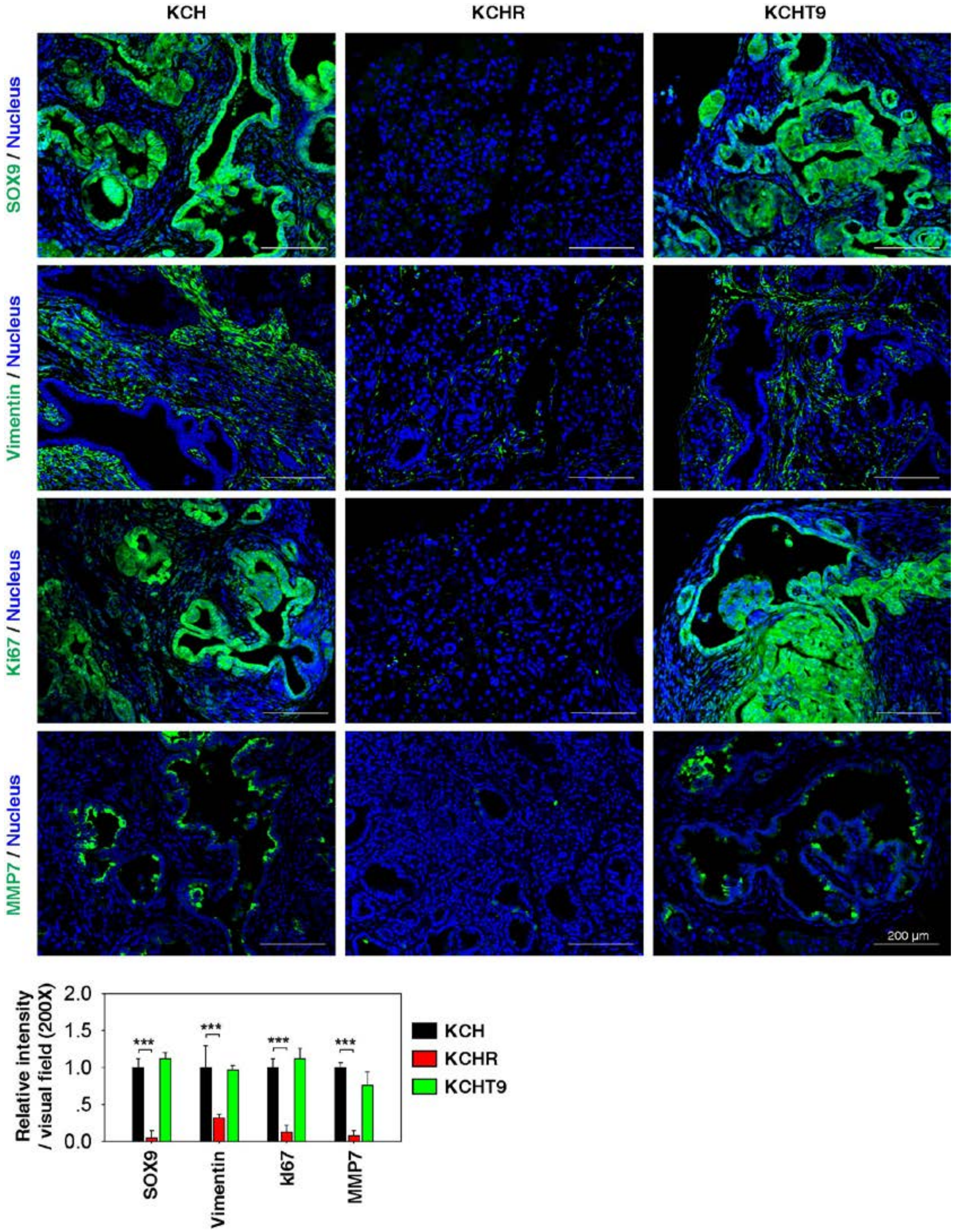

Figure S11. KCHR mice (but not KCHT9 mice) exhibited decreased expression of SOX9, vimentin, Ki-67, and MMP7 in pancreas compared with KCH mice at 12 weeks of age (n=5 mice/genotype, ***p < 0.001, data are expressed as means ± s.e.m, unpaired t-test).
