# Supplementary material for: Intracellular HMGB1 as a novel tumor suppressor of pancreatic cancer
Source: Cell Res. 2017 Apr 4;27(7):916–32. doi: 10.1038/cr.2017.51 (PMC5518983; doi:10.1038/cr.2017.51)
Supplement: Supplementary information, Figure S12 — (A) Loss of RAGE (but not TLR9) diminished recombinant nucleosomes-induced IL-6 release in isolated peritoneal macrophages and PDAC cells from indicated mice at 6 weeks of age (n=3, ***p < 0.001, data are expressed as means ± s.e.m, unpaired t-test versus KCH group). [file cr201751x12.pdf]

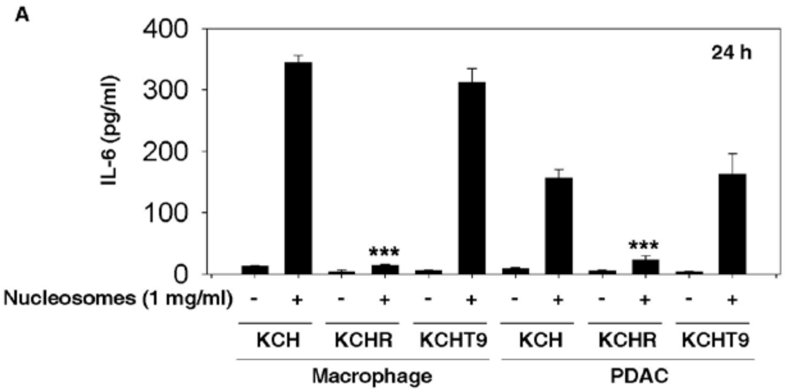

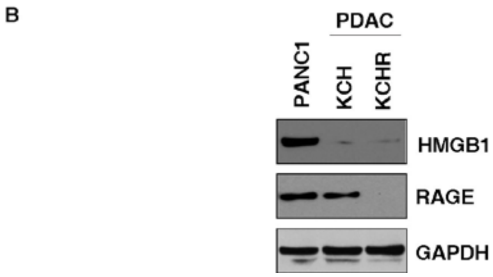

Figure S12. (A) Loss of RAGE (but not TLR9) diminished recombinant nucleosomes-induced IL-6 release in isolated peritoneal macrophages and PDAC cells from indicated mice at 6 weeks of age (n=3, ***p < 0.001, data are expressed as means ± s.e.m, unpaired t-test versus KCH group). (B) Western blot analysis of HMGB1 and RAGE expression in PDAC cells from KCH and KCHR mice. Human PANC1 cell lines were as a positive control.
