# Supplementary material for: Intracellular HMGB1 as a novel tumor suppressor of pancreatic cancer
Source: Cell Res. 2017 Apr 4;27(7):916–32. doi: 10.1038/cr.2017.51 (PMC5518983; doi:10.1038/cr.2017.51)
Supplement: Supplementary information, Table S1 — Data on tumor metastasis and invasion in KC, KCH, and KCH+/− mice [file cr201751x13.pdf]

**Table S1. Data on tumor metastasis and invasion in KC, KCH, and KCH$^{+/-}$ mice**

| No. | Strain | Sex | Age (week) | Weight loss | Metastasis/invasion |
|---|---|---|---|---|---|
| 1 | KC | F | 6 | N | N |
| 2 | KC | F | 12 | N | N |
| 3 | KC | F | 12 | N | N |
| 4 | KC | F | 24 | N | N |
| 5 | KC | M | 6 | N | N |
| 6 | KC | M | 12 | N | N |
| 7 | KC | M | 12 | N | N |
| 8 | KC | M | 24 | N | N |
| 9 | KC | M | 24 | Y | N |
| 10 | KC | M | 24 | Y | Y (lung) |
| 11 | KCH | F | 6 | Y | N |
| 12 | KCH | F | 6 | Y | N |
| 13 | KCH | F | 6 | Y | N |
| 14 | KCH | F | 6 | Y | N |
| 15 | KCH | F | 8 | Y | N |
| 16 | KCH | F | 8 | Y | Y (lung) |
| 17 | KCH | F | 12 | Y | N |
| 18 | KCH | F | 12 | Y | Y (lung, kidney) |
| 19 | KCH | F | 24 | Y | Y (lung, liver) |
| 20 | KCH | M | 6 | Y | N |
| 21 | KCH | M | 6 | Y | N |
| 22 | KCH | M | 6 | Y | N |
| 23 | KCH | M | 6 | Y | N |
| 24 | KCH | M | 6 | Y | Y (lung, liver) |
| 25 | KCH | M | 8 | Y | N |
| 26 | KCH | M | 8 | Y | Y (lung) |
| 27 | KCH | M | 8 | Y | N |
| 28 | KCH | M | 8 | Y | Y (lung) |
| 29 | KCH | M | 12 | Y | N |
| 30 | KCH | M | 12 | Y | Y (lung, liver, kidney) |

| | | | | | |
|---|---|---|---|---|---|
| 31 | KCH | M | 12 | Y | Y (liver, kidney) |
| 32 | KCH | M | 24 | Y | Y (lung, liver, kidney) |
| 33 | $KCH^{+/-}$ | F | 6 | Y | N |
| 34 | $KCH^{+/-}$ | F | 6 | Y | N |
| 35 | $KCH^{+/-}$ | F | 12 | Y | Y (lung) |
| 36 | $KCH^{+/-}$ | F | 24 | Y | Y (liver) |
| 37 | $KCH^{+/-}$ | M | 6 | Y | N |
| 38 | $KCH^{+/-}$ | M | 6 | Y | N |
| 39 | $KCH^{+/-}$ | M | 8 | Y | Y (lung) |
| 40 | $KCH^{+/-}$ | M | 24 | Y | Y (lung, liver, kidney) |

F, female; M, male; Y, yes; N, no
